# Supplementary material for: Individual and community level factors associated with anemia among children 6—59 months of age in Ethiopia: A further analysis of 2016 Ethiopia demographic and health survey
Source: PLoS One. 2020 Nov 13;15(11):e0241720. doi: 10.1371/journal.pone.0241720 (PMC7665792; doi:10.1371/journal.pone.0241720)
Supplement: S1 Annex — (DOCX) [file pone.0241720.s001.docx]

**Annex 1:** Random effects estimates of anemia for included children age 6-59 months selected from the 2016 (n=7790)

| **Estimates** | **Null mode** | **Model 1** | **Model 2** | **Model 3** |
| --- | --- | --- | --- | --- |
| Variance (δ^2^u_0_) | 0.76^*^ | 0.36^*^ | 0.28^*^ | 0.25^*^ |
| ICC (%) | 18.77 | 10.03 | 8.04 | 7.3 |
| PCV (%) | (ref) | 52.6 | 63.2 | 67 |
| Model comparison  AIC | 9967 | 8373.8 | 9692.9 | 8281 |

Key: AIC – Akaike Information Criteria, ICC - Intraclass Correlation Coefficient, PCV – Percentage Change in Variance, ^*^ p-value<0.001.
