# Supplementary material for: Individual and community level factors associated with anemia among children 6—59 months of age in Ethiopia: A further analysis of 2016 Ethiopia demographic and health survey
Source: PLoS One. 2020 Nov 13;15(11):e0241720. doi: 10.1371/journal.pone.0241720 (PMC7665792; doi:10.1371/journal.pone.0241720)
Supplement: S1 File — (PDF) [file pone.0241720.s003.pdf]

**1. melogit childanemia_coded ib(0).under_weight#ib(0).HFA1|| cluster_num:, or**

Fitting fixed-effects model:

Iteration 0:   log likelihood = -5089.5039

Iteration 1:   log likelihood = -5083.8023

Iteration 2:   log likelihood = -5083.8005

Iteration 3:   log likelihood = -5083.8005

Refining starting values:

Grid node 0:   log likelihood = -4856.3624

Fitting full model:

Iteration 0:   log likelihood = -4856.3624

Iteration 1:   log likelihood = -4854.4147

Iteration 2:   log likelihood = -4851.0847

Iteration 3:   log likelihood = -4851.0845

Mixed-effects logistic regression              Number of obs     =      7,602

Group variable:     cluster_num                Number of groups  =        636

Obs per group:

min =         1

```
                                   avg =      12.0
                                   max =        37

Integration method: mvaghermite          Integration pts.  =         7

                               Wald chi2(3)      =      55.15
Log likelihood = -4851.0845                Prob > chi2       =     0.0000
------------------------------------------------------------------------------
childanemia_coded | Odds Ratio   Std. Err.      z    P>|z|     [95% Conf. Interval]
------------------+-----------------------------------------------------------
                  |
under_weight#HFA1 |
       0#stunted  |   1.196936   .0844278     2.55   0.011     1.04239    1.374396
   1#not stunted  |   1.486241   .1798644     3.27   0.001     1.172403    1.884088
       1#stunted  |   1.638647    .113939     7.10   0.000     1.429879    1.877896
                  |
           _cons |   1.282562   .0633801     5.04   0.000     1.164165    1.412999
------------------+-----------------------------------------------------------
cluster_num       |
       var(_cons)|   .7291952   .0745652                      .5967639    .8910149
------------------------------------------------------------------------------
LR test vs. logistic model: chibar2(01) = 465.43      Prob >= chibar2 = 0.0000
```

- There is significant interaction((p>0.05) ) between child shunting and under weight
- After fitting this interaction term (under_weight#HFA1) into final model for analysis the result was not significant.

| childanemia_coded | Odds Ratio | Std. Err. | z | P>\|z\| | [95% Conf. | Interval] |
|---|---|---|---|---|---|---|
| age_coded | | | | | | |
| 24-41 | 1.827748 | .1277867 | 8.63 | 0.000 | 1.593693 | 2.096177 |
| 6-23 | 4.409101 | .443264 | 14.76 | 0.000 | 3.620557 | 5.369386 |
| under_weight#HFA1 | | | | | | |
| 1#stunted | 1.096762 | .1803339 | 0.56 | 0.574 | .7946107 | 1.513806 |
| birh_coded | | | | | | |
| larger than average | .9339993 | .0625444 | -1.02 | 0.308 | .8191183 | 1.064992 |
| smaller than average | 1.047901 | .0750079 | 0.65 | 0.513 | .9107347 | 1.205726 |

**2. melogit childanemia_coded ib(0).under_weight#ib(0).anemia_NL|| cluster_num:, or**

| childanemia_coded | Odds Ratio | Std. Err. | z | P>\|z\| | [95% Conf. Interval] | |
|---|---|---|---|---|---|---|
| under_weight#anemia_NL | | | | | | |
| 0#Anemic | 1.528909 | .1043885 | 6.22 | 0.000 | 1.33741 | 1.747828 |
| 1#Not anemic | 1.436279 | .1063553 | 4.89 | 0.000 | 1.242247 | 1.660618 |
| 1#Anemic | 2.549464 | .2566614 | 9.30 | 0.000 | 2.092937 | 3.105573 |
| _cons | 1.173312 | .0570748 | 3.29 | 0.001 | 1.066614 | 1.290682 |
| cluster_num | | | | | | |
| var(_cons) | .6025664 | .066961 | | | .4846341 | .7491966 |

LR test vs. logistic model: chibar2(01) = 329.42 Prob >= chibar2 = 0.0000

- There is significant interaction ($p<0.05$) between child under weight and maternal anemia
- After fitting this interaction term (ib(0).under_weight#ib(0).anemia_NL) into final model for analysis the result was not significant.

```
Mixed-effects logistic regression            Number of obs     =     7,069

Group variable:     cluster_num               Number of groups  =       634


                                 Obs per group:

                                       min =         1

                                       avg =      11.1

                                       max =        35


Integration method: mvaghermite               Integration pts.  =         7


                                 Wald chi2(56)     =     827.09

Log likelihood = -4082.9144                   Prob > chi2       =     0.0000

------------------------------------------------------------------------------------------

    childanemia_coded | Odds Ratio   Std. Err.      z    P>|z|     [95% Conf. Interval]

----------------------+-------------------------------------------------------------------

                      |

            age_coded |

                24-41  |   1.824768   .1275682     8.60   0.000     1.591112    2.092736

                 6-23  |   4.406734   .4428872    14.76   0.000     3.618837    5.366172

                      |

under_weight#anemia_NL |

            1#Anemic  |   1.173306   .1620526     1.16   0.247     .8950485     1.53807

                      |
```

birh_coded |

**3. melogit childanemia_coded ib(5).wealth_index#ib(0).anemia_NL || cluster_num:, or**

| childanemia_coded | Odds Ratio | Std. Err. | z | P>\|z\| | [95% Conf. Interval] | |
|---|---|---|---|---|---|---|
| wealth_index#anemia_NL | | | | | | |
| poorest#Not anemic | 1.963646 | .2042301 | 6.49 | 0.000 | 1.601525 | 2.407648 |
| poorest#Anemic | 3.274973 | .3694387 | 10.52 | 0.000 | 2.625346 | 4.085347 |
| poorer#Not anemic | 1.354231 | .1489252 | 2.76 | 0.006 | 1.091656 | 1.679963 |
| poorer#Anemic | 2.453868 | .3465919 | 6.36 | 0.000 | 1.86048 | 3.236515 |
| middle#Not anemic | 1.21929 | .1383077 | 1.75 | 0.080 | .9762313 | 1.522865 |
| middle#Anemic | 1.648055 | .2414167 | 3.41 | 0.001 | 1.236753 | 2.196143 |
| richer#Not anemic | 1.265219 | .1462374 | 2.04 | 0.042 | 1.008746 | 1.586901 |
| richer#Anemic | 1.444035 | .2312861 | 2.29 | 0.022 | 1.054978 | 1.976568 |
| richest#Anemic | 1.54458 | .22383 | 3.00 | 0.003 | 1.16268 | 2.051922 |
| | | | | | | |
| _cons | .9340817 | .0735942 | -0.87 | 0.387 | .8004249 | 1.090057 |
| cluster_num | | | | | | |
| var(_cons) | .516836 | .0603679 | | | .4110835 | .6497937 |

LR test vs. logistic model: chibar2(01) = 272.72      Prob >= chibar2 = 0.0000

- There is significant interaction((p>0.05) ) between wealth index and maternal anemia
- After fitting this interaction term (**ib(5).wealth_index#ib(0).anemia_NL**) into final model for analysis the result was not significant. So we conclude that

| childanemia_coded | Odds Ratio | Std. Err. | z | P>\|z\| | [95% Conf. Interval] | |
|---|---|---|---|---|---|---|
| | | | | | | |
| age_coded | | | | | | |
| 24-41 | 1.827772 | .1278492 | 8.62 | 0.000 | 1.59361 | 2.096341 |
| 6-23 | 4.421866 | .4446994 | 14.78 | 0.000 | 3.630795 | 5.385294 |
| | | | | | | |
| wealth_index#anemia_NL | | | | | | |
| poorest#Anemic | .9279333 | .1740177 | -0.40 | 0.690 | .6425231 | 1.340123 |
| poorer#Anemic | 1.086254 | .2313254 | 0.39 | 0.698 | .7155866 | 1.648925 |
| middle#Anemic | .7809219 | .1707509 | -1.13 | 0.258 | .508733 | 1.198741 |
| richer#Anemic | .6667046 | .1545607 | -1.75 | 0.080 | .4232541 | 1.050185 |
| | | | | | | |
| birh_coded | | | | | | |
| larger than average | .9337952 | .0625717 | -1.02 | 0.307 | .8188688 | 1.064851 |
| smaller than average | 1.046233 | .0749315 | 0.63 | 0.528 | .9092122 | 1.203904 |
| | | | | | | |
| childnum_coded | | | | | | |

**4. melogit childanemia_coded ib(0).HFA1#ib(5).wealth_index|| cluster_num:, or**

| childanemia_coded | Odds Ratio | Std. Err. | z | P>\|z\| | [95% Conf. | Interval] |
|---|---|---|---|---|---|---|
| HFA1#wealth_index | | | | | | |
| not stunted#poorest | 2.032428 | .2189457 | 6.58 | 0.000 | 1.645578 | 2.51022 |
| not stunted#poorer | 1.505907 | .179509 | 3.43 | 0.001 | 1.192153 | 1.902235 |
| not stunted#middle | 1.230027 | .148701 | 1.71 | 0.087 | .9705345 | 1.558901 |
| not stunted#richer | 1.142226 | .1399317 | 1.09 | 0.278 | .8984068 | 1.452215 |
| stunted#poorest | 2.821801 | .321505 | 9.10 | 0.000 | 2.257063 | 3.527841 |
| stunted#poorer | 1.744609 | .2169472 | 4.48 | 0.000 | 1.367252 | 2.226114 |
| stunted#middle | 1.426982 | .1917071 | 2.65 | 0.008 | 1.09664 | 1.856832 |
| stunted#richer | 1.656077 | .2416373 | 3.46 | 0.001 | 1.244177 | 2.204342 |
| stunted#richest | 1.328074 | .1824175 | 2.07 | 0.039 | 1.014625 | 1.738358 |
| _cons | .9601427 | .0787679 | -0.50 | 0.620 | .8175328 | 1.127629 |
| cluster_num | | | | | | |
| var(_cons) | .6099988 | .0665301 | | | .4925972 | .7553811 |

LR test vs. logistic model: chibar2(01) = 357.90 Prob >= chibar2 = 0.0000

| childanemia_coded | Odds Ratio | Std. Err. | z | P>\|z\| | [95% Conf. | Interval] |
|---|---|---|---|---|---|---|

| | | | | | | |
|---|---|---|---|---|---|---|
| | | | | | | |
| age_coded | | | | | | |
| 24-41 | 1.82445 | .127558 | 8.60 | 0.000 | 1.590814 | 2.092399 |
| 6-23 | 4.407824 | .4434332 | 14.75 | 0.000 | 3.61903 | 5.368541 |
| | | | | | | |
| wealth_index#HFA1 | | | | | | |
| poorest#stunted | 1.023714 | .1853824 | 0.13 | 0.897 | .7178545 | 1.459893 |
| poorer#stunted | .9570471 | .188996 | -0.22 | 0.824 | .6498895 | 1.409377 |
| middle#stunted | .9868593 | .201983 | -0.06 | 0.948 | .6607507 | 1.473916 |
| richer#stunted | 1.197104 | .2585735 | 0.83 | 0.405 | .7839221 | 1.828061 |
| | | | | | | |
