# Supplementary material for: Individual and community level factors associated with anemia among children 6—59 months of age in Ethiopia: A further analysis of 2016 Ethiopia demographic and health survey
Source: PLoS One. 2020 Nov 13;15(11):e0241720. doi: 10.1371/journal.pone.0241720 (PMC7665792; doi:10.1371/journal.pone.0241720)
Supplement: Attachment — Submitted filename: INDIVIDUAL AND COMMUNITY LEVEL FACTORS ASSOCIATED WITH ANEMIA.pdf [file pone.0241720.s006.pdf]

PLOS ONE

INDIVIDUAL AND COMMUNITY LEVEL FACTORS ASSOCIATED WITH ANEMIA AMONG CHILDREN 6 - 59 MONTHS OF AGE IN ETHIOPIA: A FURTHER ANALYSIS OF 2016 ETHIOPIA DEMOGRAPHIC AND HEALTH SURVEY

--Manuscript Draft--

**Manuscript Number:**	PONE-D-20-09803R1
**Article Type:**	Research Article
**Full Title:**	INDIVIDUAL AND COMMUNITY LEVEL FACTORS ASSOCIATED WITH ANEMIA AMONG CHILDREN 6 - 59 MONTHS OF AGE IN ETHIOPIA: A FURTHER ANALYSIS OF 2016 ETHIOPIA DEMOGRAPHIC AND HEALTH SURVEY
**Short Title:**	INDIVIDUAL AND COMMUNITY LEVEL FACTORS ASSOCIATED WITH ANEMIA AMONG CHILDREN 6 - 59 MONTHS OF AGE IN ETHIOPIA
**Corresponding Author:**	menaseb Gebrehaweria Gebremeskel, MSc<br>Adigrat University<br>Adigrat, ETHIOPIA
**Keywords:**	Anemia; children 6-59 months of age; Multilevel logistic regression
**Abstract:**	Abstract<br><br>Background: Anemia is a global public health problem; but its burden is disproportionately borne among children in the African Regions. The 2016 Ethiopia Demographic and Health Survey report showed that the prevalence of anemia among children 6-59 months of age was 57%; far exceeding the national target of 25% set for 2015. Although studies have been conducted in Ethiopia, multilevel analysis has rarely been used to identify factors associated with anemia among children. Therefore, this study aimed to identify individual and community-level factors associated with anemia among children 6-59 months of age by fitting a multilevel logistic regression model.<br>Methods : The data was obtained from the 2016 Ethiopia Demographic and Health Survey, conducted from January to June 2016, and downloaded from the website http://www.DHSprogram.com. The sample was taken using two-stage stratified sampling. In stage one, 645 Enumeration Areas and in stage two 28 households per Enumeration Area were selected. A sample of 7790 children 6-59 months of age was included. Data were analyzed using STATA version 14. A multilevel logistic regression model was fitted and an adjusted odds ratio with a 95% confidence interval was obtained.<br>Result: From the individual-level factors, anemia was associated most strongly with child age, wealth index, maternal anemia and child stunting followed by child underweight, child fever and birth order whereas from the community-level, the strongest odds of anemia occurred among children from Somali, Harari, Dire Dawa and Afar region followed by Oromia and Addis Ababa. Low community-poverty is a protective factor for anemia. The odds of anemia were 0.81 (AOR=0.81; 95% CI: 0.66, 0.99) times lower for children who were living in communities of lower poverty status than children who were living in communities of higher poverty status. Children from Somali and Dire Dawa had 3.38 (AOR=3.38; 95% CI: 3.25, 5.07) and 2.22 (AOR=2.22; 95% CI: 1.42, 3.48) times higher odds of anemia, respectively than children from the Tigray region.<br>Conclusions: This study shows that anemia among children 6-59 months of age is affected both by the individual and community level factors. It is better to strengthen the strategies of early detection and management of stunted and underweight children. At the same time, interventions should be strengthened to address maternal anemia, child fever and poverty, specifically targeting regions identified to have a high risk of anemia.
**Order of Authors:**	menaseb Gebrehaweria Gebremeskel, MSc
	Afework Mulugeta
	Abate Bekele
	Lire Lemma

Powered by Editorial Manager® and ProduXion Manager® from Aries Systems Corporation

Muzey Gebremichael
Haftay Gebremedhin
Berhe Etsay
Tesfay Tsegay
Yared Haileslasie
Yohannes Kinfe
Fre Gebremeskel
Letemichael Mezgebo
Selam Shushay

Response to Reviewers:

Responses to reviewers
Reviewer #1
Dear reviewer, thank you for your thorough review of our manuscript. Your comments are constructive and very good lessons for us. We have tried to put our responses to your constructive comments and questions. The responses are put immediately after the questions, suggestions or comments.
General comments:
The information you have analyzed and interpreted is valuable. I have made suggestions to improve the organization and presentation of your data.
1. Cut down on stating data and use the space to provide more interpretation. For example, use more graphics (ex. bar graph) showing the distribution of anemia in your strongest predictors
As per the comment, we presented the distribution of anemia by the factors found to have significant association using pie chart and bar graphs.
Review
2.Abstract
Background:
Result
2.1. Consider wording the results as Anemia was associated most strongly with ….
The result part of the abstract is reviewed and as per the comments given, we rewrite it as: Anemia was associated most strongly with child age, stunting, underweight, fever, maternal anemia, low poverty-community and Region. The odds of anemia were 0.81 AOR=0.81; 95% CI: 0.66, 0.99) times lower for children who were living in communities of lower poverty status than children who were living in communities of higher poverty status. Children from Somali and Dire Dawa had 3.38 (AOR=3.38; 95% CI: 3.25, 5.07) and 2.22 (AOR=2.22; 95% CI: 1.42, 3.48) times higher odds of anemia, respectively than children from the Tigray Region.
Conclusions
2.2. Consider using present tense throughout the paper (e.g. “shows” not showed)
The conclusion is reviewed not only in the abstract but also in the main body, and based on your comment we rewrite it as: This study shows that childhood anemia is affected both by the individual and community level factors. It is better to strengthen the strategies of early detection and management of stunted and underweight children. At the same time, interventions should be strengthened to address maternal anemia, child fever and poverty, specifically targeting regions identified to have a high risk of anemia.
2.3. Malnutrition is not specifically discussed in your results, why is it one of the main conclusion? is reviewed not only in the abstract but also in the main body
Yes. Malnutrition itself is not discussed as our result. However, we found that child stunting and underweight had strong association with anemia, and these variables are some forms of Malnutrition in children. But now your comment reminds us it is better to conclude based on our result i.e stunting and underweight. So, we rewrite the conclusion as: This study shows that childhood anemia is affected both by the individual and community level factors. It is better to strengthen the strategies of early detection and management of stunted and underweight children. At the same time, interventions should be strengthened to address maternal anemia, child fever, food insecurity and poverty, specifically targeting regions identified to have a high risk of anemia.
3. Introduction
3.1. “below” not “bellow”

The word bellow is deleted. And the sentence now becomes: Childhood anemia is defined as hemoglobin concentration in the blood below 11g/dl.
3.2. Childhood anemia is defined by age group and gender. I disagree with your definition. Anemia in children 6-59 months specifically is defined by WHO as <11g/dL hgb. Maybe that is what you meant? I would suggest stating in your methods that you defined anemia as hemoglobin <11g/dL based on xxxx definition from the WHO. For
We defined anemia in 6-59 month age children as hemoglobin <11 g/dL according to WHO criteria.
3.3. I would suggest starting with sentence on prevalence of anemia in children worldwide versus in Ethiopia, why it is important/what the complications are, then why hemoglobin is used as a measure of anemia, then the WHO definition of anemia in children 6-59 months of age, and then discuss more details on Ethiopian anemia classification and problems specific to anemia.
We carefully reviewed the introduction, and we rewrite it based on your suggestion in the revised document.
3.4. Specifically state the aims. The aims of this study are to 1), 2), 3)….
As per the comment, the aims of this study are :
1.To identify individual level factors associated with anemia among children 6-59 months of age in Ethiopia.
2.To identify community level factors associated with anemia among children 6-59 months of age in Ethiopia
4. Methods
4.1. - Where are your inclusion and exclusion criteria? What is your study population?
Based on your comment, we included the inclusion and exclusion criteria as follows. for general information, the EDHS kid record file is source of our sample, and this data contains under-five children (children 0-59 months of age).
Inclusion criteria: children 6-59 months of age who live in the selected enumeration areas (community).
Exclusion criteria: children 6-59 months of age who have no hemoglobin test result. So, according to this inclusion and exclusion criteria, we have had a total of 10,641 under-five children regardless of their hemoglobin test result. Then from these children, we exclude 1137 children less than 6 months of age (10641-1137=9504 children 6-59 months age regardless of their hemoglobin test result). Then from these children we exclude again 1714 children 6-59 months age who have no hemoglobin test result. Finally, we consider 7790 children 6-59 months age as eligible for our study.
The study population is children 6-59 months of age who were living in selected enumeration areas.
4.2. I would remove all formulas from your methods unless they are novel or controversial methods of analysis.
As mentioned in the method section of the document, the analysis method we applied is multilevel binary logistic regression analysis. These multilevel models are different from the ordinary single level logistic regression in such a way that they can handle hierarchical nature of data like that of the Demographic and Health Survey (DHS) data. Recently, these models are becoming popular models. However, in our country Ethiopia, these models are still rarely used. That is why we preferred to include their formula in our document.
Data Source
4.3. I would again spell out EDHS the first time in this section, but that is up to you.
EDHS is abbreviation for the Ethiopia Demographic and Health Survey and mostly it is the name given for the respective Ethiopia Demographic Health Surveys conducted in Ethiopia.
4.4. Which MOH? Specify Ethiopian Ministry of Health.
We correct it and the sentence becomes “The 2016 EDHS is the fourth survey which is implemented by the Central Statistical Agency (CSA) in collaboration with the Ethiopian Ministry of Health under the technical assistance of International Classification of Functioning, Disability, and Health (ICF) thorough the DHS Program”.
4.5. Yes, we need to know that you have permission to access the EDHS database, but I don’t think we need to know how you obtained permission.
Our intention to write how we obtained permission to access the EDHS data was just to convince readers of this paper that we are legal. But based on your comment we removed the sentences how we obtained permission.
4.6. “through” not “thorough”
We correct it.
4.7. Study Design and Sample Size. The cross sectional survey you describe is the

original method of the EDHS data collection? Or are we talking about your study?? If this is your study, then very briefly describe the methods of the EDHS in the first paragraph.
The cross sectional survey we described in our document is the original method of the EDHS data collection. We did nothing on the study design and sampling design just we used the original method of the EDHS data collection methods.
4.8. We need your inclusion and exclusion criteria in order to understand why it is important that 9504 children were 6-59 months of age.
Inclusion criteria: children 6-59 months of age who live in the selected enumeration areas (community).
Exclusion criteria: children 6-59 months of age who have no hemoglobin test result. So, according to this inclusion and exclusion criteria, we have had a total of 10,641 under-five children regardless of their hemoglobin test result. Then from these children, we exclude 1137 children less than 6 months of age (10641-1137=9504 children 6-59 months age regardless of their hemoglobin test result). Then from these children we exclude again 1714 children 6-59 months age who have no hemoglobin test result. Finally, we consider 7790 children 6-59 months age as eligible for our study".
4.9. Revise grammar for the sentence "the 2016 EDHS was used…"
We corrected it as: The 2016 EDHS had used a stratified two-stage cluster sampling design.
4.10. Study Variables (Maybe a better title is Definitions?)
We amend it as: Definitions of study variables
4.11. What do you mean the variables were selected from the literature?
During the conception of our study, we reviewed literatures just to know what was done before regarding anemia among children 6-59 months age and what is left to solve the problem. So, during this we observed and identified variables included in the literatures done before. Bay the way, we include also new variables for the first time in our study.
4.12. I would not go to the extent of telling us if your variables are dichotomous.
Yes you are right. But we don't think that all readers have the same knowledge in understanding the outcome variable (anemia) whether it is binary variable. That is why we indicated as it is dichotomous.
4.13. Perhaps simplify this paragraph by first stating that "We assessed the impact of individual and community-level variables on our primary outcome of anemia. We defined anemia as … (see below). Individual level variables were …. Community-level variables were ….."
We reviewed this paragraph and based on your comment, we rewrite it as:
We assessed the impact of individual and community-level variables on anemia among children 6-59 months of age. We defined anemia in 6-59 month children as hemoglobin (hg) <11 g/dL according to WHO criteria. Individual level variables were: sex, age, birth order, birth weight, religion, number of under-five children, childhood wasting, underweight, stunting, symptoms of acute respiratory infection, child fever and diarrhea, maternal anemia and age, parents' educational and employment status, wealth index, source of drinking water, and type of toilet facility, whereas community-level variables were: Region, community-poverty, community-women education and community- women unemployment"
4.14. "We defined anemia in 6-59 month children as <11 g/dL according to WHO criteria (reference)." Please see earlier comments. Remember that unless you specify, I don't even know that you are discussing children 6-59 months only.
We corrected it as per your comment as" We defined anemia in 6-59 month age children as hemoglobin <11 g/dL according to WHO criteria".
4.15. Definitions are important. Consider using full sentences such as, "Wealth Index is a composite measure…" instead of : .
As per the comments you provided, we rewrite it as "Wealth index: is a composite measure of a household's cumulative living standard. It was calculated based on household ownership of selected assets such as televisions and bicycles, cars; materials used for the housing construction; source of drinking water; and type of sanitation facilities. It was then generated using principal components analysis and the individual households were placed on a continuous scale of relative wealth. In the EDHS all mothers and children were assigned a standardized wealth index score. It was measured as a composite variable made up of five quintiles as poorest, poorer, middle, richer and richest.
4.16. Consider combining anthropometrics into a paragraph "Anthropometrics: Stunting was defined as height or length for age, HFA) <-2, wasting as ….
We corrected it as "Anthropometrics: Stunting was defined as height or length for age,

(HFA) <-2SD (standard deviation), wasting as weight-for-height (WFH) <-2 SD and underweight as weight-for-age (WFA) <-2 SD.
4.17. What about children without a mother? Are they included? I wonder if some of the "mothers" are other relatives who are the child's guardian.
Yes. We include children without a mother that is children with their guardian or caretakers. So the whole document is corrected by considering this comment.
4.18. What do you mean by "poor" or "poorest" etc.? Are these economic quintiles? Please define objectively this subjective term. This makes it difficult for me to interpret Figure 2.
Poor or poorest are economic quintiles. In the 2016 Ethiopia Demographic and Health Survey, these are quintiles of wealth index (economic quintiles) calculated based on household ownership of selected assets such as televisions and bicycles, cars; materials used for the housing construction; source of drinking water; and type of sanitation facilities. It was then generated using principal components analysis and the individual households were placed on a continuous scale of relative wealth. In the EDHS all mothers (caretakers) and children were assigned a standardized wealth index score. It was measured as a composite variable made up of five quintiles as poorest, poorer, middle, richer and richest
Methods of data analysis
4.19. I would cut this section down to two paragraphs. The explanation of your model also needs to be condensed to one or two paragraphs. You may consider sharing your variable selection as an Appendix or supplement.
We aimed to explain more about the models to our reader, since it is unusual model especially in our country. But we annexed Table 1 as "annex 1" at the end of the main document.
5. Results
5.1. How many children were included?
We included 7790 children 6 -59 months of age who have hemoglobin test result
5.2. Please first state how many children you included and basic demographics (age, gender). Then state prevalence of anemia. Then go on to other variables.
We accepted the comment and we rewrite it again as" Seven thousand seven hundred ninety (7790) children 6 -59 months of age were included in this study. Above half (52%) of the children were male, and 34.2%, 33.3% and 32.2% where in the age category of 6-23, 24-41 and 42-59 months of age, respectively with mean ± SD (standard deviation) of 32 +15 months. The prevalence of anemia was 57.6% with a median hemoglobin concentration of 10.7 (IQR: 9.6-11.6)".
5.3. The sentence tells me that children were living with their partners. Surely this is not what is meant. Consider using "caretakers" to clarify when you discuss characteristics of the caretaker or mother.
We accepted the comment, and we rearranged the sentence as" Almost all of the respondents mothers (caretakers) (95%) were living with their respective partners and most of them were Muslims (40%) followed by Orthodox Christians (34%).
5.4. I would be more interested in seeing anemia sorted by economic status than a pie chart only of economic status. I think seeing child anemia sorted by maternal anemia (what is the definition of this, by the way?) would also provide useful information, as maternal anemia is a known risk factor for childhood anemia especially for the youngest.
All the significant variables have sorted by anemia and this is indicated in table 4. Maternal anemia is defined as hemoglobin less than or equal to 11 g/dL (hg<=11 g/dL) according to WHO criteria. In the EDHS, hemoglobin levels were adjusted for pregnancy because during pregnancy the increase in maternal blood volume and the iron needs of the fetus decreases the blood Hb level.
5.5. Table 2. I think this should be Table 1. I would not present the current Table 1. Good to have this table (2), but some of this information can be presented as supplemental data. Please indicate the units, for example child age (months). Please consider labeling this as something like "Table x. Demographic characteristics of included children age 6-59 months selected from the 2016 EDHS."
We annexed table 1, and we rename Table 2 as Table 1. We accept all the other comments and we amend our document accordingly.
5.6. Why does birth order 4-5 matter more than >=6?
According to our result presented in Table 4, birth order >=6 matter more than birth order 4-5. The odds of anemia were 1.26 (AOR=1.26; 95% CI: 1.00, 1.61) times higher for children with birth order six and above than first-order children (Table 4).
5.7. Community Level characteristics table: Please note this is mislabeled as Table 1.

Powered by Editorial Manager® and ProduXion Manager® from Aries Systems Corporation

Please also see above suggestion for the title.
We corrected it accordingly.
Individual and community-level factors associated with anemia
5.8. Consider presenting the odds ratios in Table 1 next to the demographics. This would condense a lot of information into less space. Alternatively, cut down your demographics to the essential for Table 1 (currently labeled 2) and then keep Table 4 as is. Then present some of your data in graphs as well.
Table 1 (currently labeled 2) has presented simple frequency of all included variables. In addition, this table did not show any distribution of anemia by these factors, whereas Table 4 in the second column to right shows the anemia distribution by the significant factors. Then next to this, the Adjusted OR is written with their p-values. Therefore, these two tables are independent.
5.9. Consider instead of simply stating all the OR, giving some context of importance. For example, “the strongest odds of anemia occurred with x and y (data).” Or “anemia was most strongly associated with ….”
We accepted the comment. we rewrite it as “From the individual-level factors, anemia was most strongly associated with child age, wealth index, maternal anemia and child stunting, whereas from the community-level, the strongest odds of anemia occurred among children from Somali, Harari, Dire Dawa and Afar region (Table 4).
5.10. Table 4: I would remove crude OR and present the adjusted OR with p value in the next column. Also, see above to cut down on demographics table as much of the same information is repeated here. Please explain what you adjusted for. Tell me more about the results of your multivariate model? Which variables made it into your model? This is not clear.
We removed crude OR and we present the adjusted OR with p value in the next column.
The demographic information written in Table 4 is not the same as the demographic information written in Table 1. Table 1 (previously labeled as Table 2) has presented simple frequency of all included variables. In addition, this table did not show any distribution of anemia by these factors, whereas Table 4 in the second column to right shows the anemia distribution by the significant factors. Then next to this, the Adjusted OR is written with their p-values.
5.11. Please explain what you adjusted for. Tell me more about the results of your multivariate model?
First bivariate analysis was performed to see the effect of each predictor variable on the outcome variable using a significance level of p<0.25 independently. Accordingly, anemia among children aged 6-59 months was associated with number of <5 children in the household, parents educational level and employment status, child age, religion of mother, birth order, maternal age, type of toilet facility , source of drinking water, wealth index, child stunting, wasting and underweight, fever, diarrhea, child deworming, symptoms acute respiratory infection, maternal anemia status, women community-education , place of residence, community-poverty , region and community-women unemployment status. Then all this variables were included simultaneously in to the final model to estimate adjusted odds ratios with 95% Confidence Interval (CI) at a significance level of p<0.05.
In short, in the multivariable analysis the following variables were adjusted and controlled.
1.number of <5 children in the household
2.child age
3. religion of mother
4.birth order
5. maternal and husband employment status
6.maternal age, type of toilet facility
7. source of drinking water
8.wealth index
9.child stunting, wasting, underweight, fever, diarrhea
10.child deworming, acute respiratory infection
11.maternal anemia status
12. community- women education
13. place of residence, community-poverty
14. region
15.community- women unemployment
After adjusting for all this variables, we found that anemia was most strongly associated with child age, wealth index, maternal anemia, child stunting, underweight,

Powered by Editorial Manager® and ProduXion Manager® from Aries Systems Corporation

	child fever, birth order, community-poverty and region (Somali, Harari, Dire Dawa, Afar, Oromia, Addis Ababa, Amhara and Benishangul). The result in<br>Table 4 shows only the significant variables found at the multivariable analysis.<br>6. Discussion<br>6.1. “This study aimed to ….” (not was aimed to)<br>We correct it as” This study aimed to identify individual and community-level factors associated with anemia among children aged 6-59 months”.<br>6.2.“This finding indicated that” is too specific to one finding when you are discussing an entire study. Consider wording like: “We found that anemia was most strongly associated with … “ or “We found that … were the strongest predictors of anemia followed by…”<br>We accepted the comment and we rearranged as” We found that anemia among children aged 6-59 was most strongly associated with individual-level factors such as child age, wealth index, maternal anemia and child stunting followed by child underweight, child fever and birth order, whereas from the community-level the strongest odds of anemia occurred among children from Somali, Harari, Dire Dawa and Afar region followed by Oromia and Addis Ababa”.<br>6.3. Consider making a figure using a flowchart of the strongest predictors of anemia (from your model) with the adjusted OR or other information along the arrows that lead to anemia. This would be an excellent visual representation of your most important conclusion.<br>We corrected based on the, comment that order the strongest predictor in text, you provided to us. But the comments consider making a figure using a flowchart of the strongest predictors of anemia is not clear, sorry.<br>6.4. What about confounders or collinearity? How confident are you that those factors did not influence your model?<br>We have checked confounders or collinearity to our model and no confounders or collinearity was detected.<br>7. Conclusion<br>7.1. I would emphasize here the strongest of all predictors first.<br>We found that anemia among children aged 6-59 was most strongly associated with individual-level factors such as child age, wealth index, maternal anemia and child stunting followed by child underweight, child fever and birth order, whereas from the community-level the strongest odds of anemia occurred among children from Somali, Harari, Dire Dawa and Afar region followed by Oromia and Addis Ababa.<br>7.2. I think going from economic quartile as a risk factor to suggesting food subsidies is a big stretch. Perhaps say something like “our model predicts that … are most strongly associated with anemia in Ethiopian children 6-59 months with additional contribution from …. This suggests that interventions targeted to the improvement of …. may contribute to a reduction in childhood anemia and its devastating complications.”<br>Our model predicts that child age, maternal anemia and child stunting followed by child underweight, child fever and birth order and region are most strongly associated with anemia in Ethiopian children 6-59 months with additional contribution from wealth index. This suggests that interventions targeted to the improvement of Economic subsidy may contribute to a reduction in childhood anemia and its devastating complications.<br>Reviewer #2<br>Dear reviewer, thank you for your thorough review of our manuscript and for encouraging us.
**Additional Information:**	
**Question**	**Response**
**Financial Disclosure**<br><br>Enter a financial disclosure statement that describes the sources of funding for the work included in this submission. Review the submission guidelines for detailed requirements. View published research articles from *PLOS ONE* for specific examples.	The author(s) received no specific funding for this work

This statement is required for submission and **will appear in the published article** if the submission is accepted. Please make sure it is accurate.<br><br>**Unfunded studies**<br>Enter: *The author(s) received no specific funding for this work.*<br><br>**Funded studies**<br>Enter a statement with the following details:<br>• Initials of the authors who received each award<br>• Grant numbers awarded to each author<br>• The full name of each funder<br>• URL of each funder website<br>• Did the sponsors or funders play any role in the study design, data collection and analysis, decision to publish, or preparation of the manuscript?<br>• **NO** - Include this sentence at the end of your statement: *The funders had no role in study design, data collection and analysis, decision to publish, or preparation of the manuscript.*<br>• **YES** - Specify the role(s) played.<br><br>* typeset	
**Competing Interests**<br><br>Use the instructions below to enter a competing interest statement for this submission. On behalf of all authors, disclose any competing interests that could be perceived to bias this work—acknowledging all financial support and any other relevant financial or non-financial competing interests.<br><br>This statement **will appear in the published article** if the submission is accepted. Please make sure it is accurate. View published research articles from *PLOS ONE* for specific examples.	The authors have declared that no competing interests exist

Powered by Editorial Manager® and ProduXion Manager® from Aries Systems Corporation

**NO authors have competing interests**<br><br>Enter: *The authors have declared that no competing interests exist.*<br><br>**Authors with competing interests**<br><br>Enter competing interest details beginning with this statement:<br><br>*I have read the journal's policy and the authors of this manuscript have the following competing interests: [insert competing interests here]*<br><br>* typeset	
**Ethics Statement**<br><br>Enter an ethics statement for this submission. This statement is required if the study involved:<br><br>• Human participants<br>• Human specimens or tissue<br>• Vertebrate animals or cephalopods<br>• Vertebrate embryos or tissues<br>• Field research<br><br>Write "N/A" if the submission does not require an ethics statement.<br><br>General guidance is provided below. Consult the submission guidelines for detailed instructions. **Make sure that all information entered here is included in the Methods section of the manuscript.**	Ethical approval was obtained from Mekelle University, College of Health Sciences Ethical Review Committee (ERC). Approval to access the 2016 EDHS dataset was obtained from DHS Program, after making a request via DHS program website and the data was used for analysis purpose only.

Powered by Editorial Manager® and ProduXion Manager® from Aries Systems Corporation

<table>
<tr><td>Format for specific study types

Human Subject Research (involving human participants and/or tissue)
• Give the name of the institutional review board or ethics committee that approved the study
• Include the approval number and/or a statement indicating approval of this research
• Indicate the form of consent obtained (written/oral) or the reason that consent was not obtained (e.g. the data were analyzed anonymously)

Animal Research (involving vertebrate animals, embryos or tissues)
• Provide the name of the Institutional Animal Care and Use Committee (IACUC) or other relevant ethics board that reviewed the study protocol, and indicate whether they approved this research or granted a formal waiver of ethical approval
• Include an approval number if one was obtained
• If the study involved non-human primates, add additional details about animal welfare and steps taken to ameliorate suffering
• If anesthesia, euthanasia, or any kind of animal sacrifice is part of the study, include briefly which substances and/or methods were applied

Field Research

Include the following details if this study involves the collection of plant, animal, or other materials from a natural setting:
• Field permit number
• Name of the institution or relevant body that granted permission</td><td></td></tr>
<tr><td>Data Availability

Authors are required to make all data underlying the findings described fully available, without restriction, and from the time of publication. PLOS allows rare exceptions to address legal and ethical concerns. See the PLOS Data Policy and FAQ for detailed information.</td><td>Yes - all data are fully available without restriction</td></tr>
</table>

A Data Availability Statement describing where the data can be found is required at submission. Your answers to this question constitute the Data Availability Statement and **will be published in the article**, if accepted.<br><br>**Important:** Stating 'data available on request from the author' is not sufficient. If your data are only available upon request, select 'No' for the first question and explain your exceptional situation in the text box.<br><br>Do the authors confirm that all data underlying the findings described in their manuscript are fully available without restriction?	
**Describe where the data may be found in full sentences. If you are copying our sample text, replace any instances of XXX with the appropriate details.**<br><br>• If the data are **held or will be held in a public repository**, include URLs, accession numbers or DOIs. If this information will only be available after acceptance, indicate this by ticking the box below. For example: *All XXX files are available from the XXX database (accession number(s) XXX, XXX.*).<br>• If the data are all contained **within the manuscript and/or Supporting Information files**, enter the following: *All relevant data are within the manuscript and its Supporting Information files.*<br>• If neither of these applies but you are able to provide **details of access elsewhere**, with or without limitations, please do so. For example:<br><br>*Data cannot be shared publicly because of [XXX]. Data are available from the XXX Institutional Data Access / Ethics Committee (contact via XXX) for researchers who meet the criteria for access to confidential data.*<br><br>*The data underlying the results presented in the study are available from (include the name of the third party*	All relevant data are within the manuscript and its Supporting Information files.

and contact information or URL).<br>• This text is appropriate if the data are owned by a third party and authors do not have permission to share the data.<br><br>* typeset	
Additional data availability information:	Tick here if the URLs/accession numbers/DOIs will be available only after acceptance of the manuscript for publication so that we can ensure their inclusion before publication.

Powered by Editorial Manager® and ProduXion Manager® from Aries Systems Corporation

Manuscript Click here to access/download;Manuscript;Manuscript.docx

INDIVIDUAL AND COMMUNITY LEVEL FACTORS ASSOCIATED WITH ANEMIA AMONG CHILDREN 6 - 59 MONTHS OF AGE IN ETHIOPIA: A FURTHER ANALYSIS OF 2016 ETHIOPIA DEMOGRAPHIC AND HEALTH SURVEY

Menaseb Gebrehaweria[1*], Afework Mulugeta[2], Abate Bekele[3], Lire Lemma[4], Muzey Gebremichael[1], Haftay Gebremedhin[1], Berhe Etsay[1], Tesfay Tsegay[5], Yared Haileslasie[5], Yohannes Kinfe[6], Fre Gebremeskel[1], Letemichael Mezgebo[1], Selam Shushay[7]

1 Department of Public Health, College of Medicine and Health Sciences, Adigrat University, Tigray, Ethiopia

2 Department of Nutrition, School of Public Health, College of Health Sciences, Mekelle University, Tigray, Ethiopia,

3 Department of Biostatistics, School of Public health, College of Health Sciences, Mekelle University, Tigray, Ethiopia

4 Department of Public Health, College of Medicine and Health Sciences, Wachamo University, Sothern Nations Nationalities and Peoples of Ethiopia

5 Department of Nursing, College of Medicine and Health Sciences, Adigrat University, Tigray, Ethiopia

6 Department of Public Health, College of Medicine and Health Sciences, Aksum University, Tigray, Ethiopia

7 Department of Midwifery, College of Medicine and Health Sciences, Adigrat University, Tigray, Ethiopia

[1*] **Email-menasebgh@gmail.com**

Abstract

Background: Anemia is a global public health problem; but its burden is disproportionately borne among children in the African Regions. The 2016 Ethiopia Demographic and Health Survey report showed that the prevalence of anemia among children 6-59 months of age was 57%; far exceeding the national target of 25% set for 2015. Although studies have been conducted in Ethiopia, multilevel analysis has rarely been used to identify factors associated with anemia among children. Therefore, this study aimed to identify individual and community-level factors associated with anemia among children 6-59 months of age by fitting a multilevel logistic regression model.

Methods: The data was obtained from the 2016 Ethiopia Demographic and Health Survey, conducted from January to June 2016, and downloaded from the website http://www.DHSprogram.com. The sample was taken using two-stage stratified sampling. In stage one, 645 Enumeration Areas and in stage two 28 households per Enumeration Area were selected. A sample of 7790 children 6-59 months of age was included. Data were analyzed using STATA version 14. A multilevel logistic regression model was fitted and an adjusted odds ratio with a 95% confidence interval was obtained.

Result: From the individual-level factors, anemia was associated most strongly with child age, wealth index, maternal anemia and child stunting followed by child underweight, child fever and birth order whereas from the community-level, the strongest odds of anemia occurred among children from Somali, Harari, Dire Dawa and Afar region followed by Oromia and Addis Ababa. Low community-poverty is a protective factor for anemia. The odds of anemia were 0.81 (AOR=0.81; 95% CI: 0.66, 0.99) times lower for children who were living in communities of lower poverty status than children who were living in communities of higher poverty status. Children from Somali and Dire Dawa had 3.38 (AOR=3.38; 95% CI: 3.25, 5.07) and 2.22 (AOR=2.22; 95% CI: 1.42, 3.48) times higher odds of anemia, respectively than children from the Tigray region.

Conclusions: This study shows that anemia among children 6-59 months of age is affected both by the individual and community level factors. It is better to strengthen the strategies of early detection and management of stunted and underweight children. At the same time,

interventions should be strengthened to address maternal anemia, child fever and poverty, specifically targeting regions identified to have a high risk of anemia.

Introduction

Globally, anemia affects around 800 million children and women; of which 42.6% are children (1). African countries have the highest prevalence (62.3%) followed by South-East Asia (53.8%) and Eastern Mediterranean Region (48.6%) (1). Furthermore, it is a major public health problem in Sub-Saharan African countries with much national prevalence estimated to be above 40% (2).

According to the 2011 Ethiopia Demographic and Health Survey (EDHS), anemia prevalence among children 6-59 months of age was 44% (3). In addition, the 2016 EDHS report showed that prevalence of anemia among children 6-59 months of age was 57% (4). This indicates that anemia is a severe public health problem which increased by 13% within five years period, during a period when the government of Ethiopia undertook efforts such as vitamin 'A' supplementation, deworming, and use of fortified foods to reduce anemia through national nutrition programs (5).

Anemia is an important indicator of poor nutrition and health with major consequences of socioeconomic development (6). Children younger than two years of age with severe anemia are at increased risk of mortality and, even mild forms, which might be corrected cause permanent cognitive damage by decreasing attention span and shortening of memory (7).

Although biochemical and hematological tests exist, hemoglobin concentration in the blood is the most reliable indicator of anemia at the population level (8). This latter method was used to assess for anemia among children aged 6-59 months in the 2016 Ethiopia Demographic Health Survey (EDHS) (4).

According to the WHO criteria, anemia in children 6-59 months of age is defined as hemoglobin concentration in the blood below 11g/dl (9). Anemia is said to be a severe public health problem when its prevalence is 40% or more, a moderate public health problem when its prevalence is between 20 and 40% and a mild public health problem when its prevalence is between 5 and 20% in any group (10).

In Ethiopia, previous studies have linked anemia to factors such as child age, child nutritional status, parents' educational level, and wealth index (11-15). However, almost all studies used single-level analysis techniques with population groups localized in a specific study area (11, 12, 14, 15). The Single-level analysis assumes that there is no community effect beyond the characteristics of individuals (16). That is, the impact of community-level factors on anemia among children aged 6-59 months remains under-studied. Moreover, analyzing hierarchical data like the DHS using single-level analysis leads to incorrect estimation of parameters and standard errors (17).

Using multilevel analysis technique, community-level effects can be identified from individual-level effects (18-21). However, this approach has rarely been used in Ethiopia to identify factors associated with anemia among children. One study that used multilevel-analysis technique failed to examine the effect of some factors such as poor nutritional status of children (stunting, wasting and underweight), child health-related factors (fever, diarrhea and respiratory infection), maternal anemia, and variables aggregated at the community level (18).

Finally, the aims of this study were:

1. To identify individual level factors associated with anemia among children 6-59 months of age in Ethiopia.
2. To identify community level factors associated with anemia among children 6-59 months of age in Ethiopia.

Methods and Materials

Data source

Data were extracted from the nationally representative 2016 EDHS. The 2016 EDHS is the fourth survey which is implemented by the Central Statistical Agency (CSA) in collaboration with the Ethiopian Ministry of Health under the technical assistance of International Classification of Functioning, Disability, and Health (ICF) through the DHS Program. Ethical approval was obtained from Mekelle University, College of Health Sciences Ethical Review Committee (ERC). Approval to access the 2016 EDHS data set was obtained from DHS Program, after making a request via DHS program website (http//www.DHSprogram.com). The EDHS data has no individual identifiers which could affect the confidentiality of participants and the data was used for analysis purpose only.

Study population: children 6-59 months of age who were living in selected enumeration areas.

Inclusion and exclusion criteria

- ✓ Inclusion criteria: children 6-59 months of age who live in the selected enumeration areas (community).
- ✓ Exclusion criteria: children 6-59 months of age who have no hemoglobin test result.

Study design and sample size

A population-based cross-sectional survey was used to collect the 2016 EDHS data. The 2016 EDHS had used a stratified two-stage cluster sampling design. Stratification was achieved by separating each region into urban and rural areas, yielding 21 sampling strata. In the first stage, 645 Enumeration Areas (EAs) or clusters were selected. Among the selected 645 EAs, 202 were in urban and 443 in rural areas. In the second stage, households were the sampling units and a fixed number of 28 households per each EA were selected. From the total of 10,641 under-five years old children, 9504 were children 6-59 months of age. Data on hemoglobin level from the survey were available for 7790 children.

Definitions of study variables

We assessed the impact of individual and community-level variables on anemia among children 6-59 months of age. We defined anemia in 6-59 month age children as hemoglobin <11 g/dL

according to WHO criteria (8). Individual level variables were: sex, age, birth order, birth weight, religion, number of under-five children, childhood wasting, underweight, stunting, symptoms of acute respiratory infection, child fever and diarrhea, maternal anemia and age, parents' educational and employment status, wealth index, source of drinking water, and type of toilet facility, whereas community-level variables were: region, community-poverty, community-women education and community- women unemployment.

Wealth index is a composite measure of a household's cumulative living standard. It was calculated based on household ownership of selected assets such as televisions and bicycles, cars; materials used for the housing construction; source of drinking water; and type of sanitation facilities. It was then generated using principal components analysis and the individual households were placed on a continuous scale of relative wealth. In the EDHS all mothers and children were assigned a standardized wealth index score. It was measured as a composite variable made up of five quintiles as poorest, poorer, middle, richer and richest (4).

Anthropometrics: stunting was defined as height or length for age (HFA) <-2SD (standard deviation), wasting as weight-for-height (WFH) <-2 SD and underweight as weight-for-age (WFA) <-2 SD.

The community-level variables that directly measure the community characteristics in the 2016 EDHS were the place of residence (rural or urban) and region (either of the nine regions or the two administrative cities). We created also other additional variables by aggregating the individual level's characteristics within their respective clusters. These variables were: community poverty, community women education and community women unemployment.

Community-poverty is the proportion of mothers who reside in poor or poorest households in the community. The aggregate of the poorest or poor individuals can show the overall poverty of the cluster. For this proportion, the median value was calculated as summary statistics and categorized as 'High' or 'Low' based on this national median value.

Community-women education (CWE) is proportion of mothers aged 15–49 with secondary or higher education in the community. The median value was calculated as summary statistics that represent the educational status of women in the cluster and was categorized as 'High' or 'Low' based on the national median value.

Community-women unemployment status (CWUe) is proportion of mothers aged 15–49 who were not employed in the community in the past twelve months. It was categorized as high if clusters had more than or equal to the national median proportion of unemployed mothers or low otherwise.

Methods of data analysis

Before doing any analysis, weighting and normalization were done for the sample in order to ensure the representativeness of the sample to different regions and their place of residence. Data were analyzed by Stata version 13 and a multilevel binary logistic regression model was fitted. Frequencies, percentages, graphs and charts were used to describe categorical variables. The effect of each predictor variable on the outcome variable was checked at a significance level of $p \leq 0.25$ independently (22). Variables that are statistically significant at the bivariate multilevel logistic regression analysis were considered as candidates for multivariable analysis. Accordingly, in the multivariable analysis the following variables were adjusted and controlled: number of <5 children in the household, child age, religion of mother (caretaker), birth order, parents employment status and educational level, maternal age, type of toilet facility, source of drinking water, wealth index, child stunting, wasting, underweight, fever, diarrhea, child deworming, symptoms of acute respiratory infection, maternal anemia, community- women education, place of residence, community-poverty, region and community- women unemployment. Adjusted Odds Ratio (AOR) with 95% Confidence Interval (CI) at a significance level of $p<0.05$ was estimated. The result of multivariable analysis for individual and community-level factors associated with anemia among children aged 6-59 months is shown in Table 4.

Assuming varying intercepts across communities (clusters) but fixed coefficients, four models were developed. The first one was the null model which is fitted without independent variables; this was used to determine the variance in anemia status between the clusters in the sample. Whereas, model one was adjusted for individual-level factors and used to examine their contribution to the variation of anemia status. Model two was adjusted for community level factors and was used for examining whether the community-level variables explain between-cluster variation on childhood anemia. Model three was developed by combining both the individual and community level variables.

Null model: for individual *i* in community j, the model can be represented as**:**

$$Y_{ij} = \Upsilon_{00} + u_{0j} + \varepsilon_{ij} \text{ .... null model (17) (23)}$$

Where:

Y_{ij} is anemia status of ith child in the jth cluster

Υ_{00} = is the intercept; that is the probability of having anemia in the absence of explanatory variables

u_{0j} = community-level effect; ε_{ij} error at individual level

Mixed model

This model was derived by mixing both individual and community level factors simultaneously.

$$Y_{ij} = \Upsilon_{00} + \Upsilon_{k0}X_{kij} + \Upsilon_{0p}z_{pj} + u_{0j} + \varepsilon_{ij} \text{ ......(24)}$$

Where: The term γ_{k0} is the regression coefficient of the individual-level variable X_k and γ_{0p} is the regression coefficient of the community-level variable Z_p. X_k and Z_p were individual and community-level explanatory variables respectively. The subscripts *i* and *j* represent for the individual level and cluster number respectively.

Intraclass correlation coefficient (ICC): a measure of within-cluster homogeneity and the proportion of variance due to between-cluster differences.

$$ICC = \frac{\delta^2{}_{u_0}}{\delta^2{}_{u_0} + \pi^2/3}$$

Where: $\delta^2{}_{u_0}$ = between cluster (community) variances and $\frac{\pi^2}{3}$ = with in cluster (community) variance. The value of $\frac{\pi^2}{3}$ in case of standard logistic distribution is 3.29 (25) .

The null model showed that there was a significant variation in anemia status among clusters ($\delta^2{}_{u0}$=0.76, p-value<0.001). The ICC was 18.77%, meaning that 18.77% of the total variability in odds of anemia was due to between community differences or attributable to the unobserved factors either at community-level or at individual-level. This indicates that using a multilevel logistic regression model is better for getting valid estimates than single-level logistic regression.

The variance which was due to the clustering effect decreased from 18.77% in the null model to 10.03%, 8.04% , and 7.03% in model one, model two and model three, respectively (Annex 1).

Proportional change in variance (PCV): calculated with reference to the null model to see relative contribution of factors to explain variation in childhood anemia.

PCV= ($\frac{V0-V1}{V0}$)*100 Where: V_0 is between community variance in the null model; V_1 is between community variance in the consecutive models (17).

Model three had the highest PCV which is 67%. This shows that 67% of the variance in the anemia status among children was due to the simultaneous effect of both individual and community-level factors found in the model (Annex 1).

Model diagnostics and adequacy checking

Multicollinearity was checked by using a variation inflation factor (VIF) with a cut-off point of 10 (26). It was checked for the independent variables in the final model and the VIF was found to range from 1.2 to 4.2 with mean VIF of 2.1. This shows that multicollinearity might not be a problem. Interaction effect was examined by fitting regression models that contained cross-product terms that yield no significant ($p>0.05$) interaction effect. Model selection was carried out by using Akaike information criteria (AIC). AIC values for each model were compared and the model with the lowest value of AIC was considered as a better explanatory model (25). Accordingly, model 4 with AIC value of 8281 was selected as the best model for explaining anemia status among children aged 6-59 months in Ethiopia (Table 1). Model accuracy was checked by the area under the curve. The Receiver Operating Characteristics curve (ROC-curve) provides a measure of the model’s ability to discriminate between those subjects who experience the outcome of interest versus those who do not (27). The area under the ROC of this model was 0.7376; which means the ROC curve accuracy for outcome variable (anemia) was 74% (Fig 1).

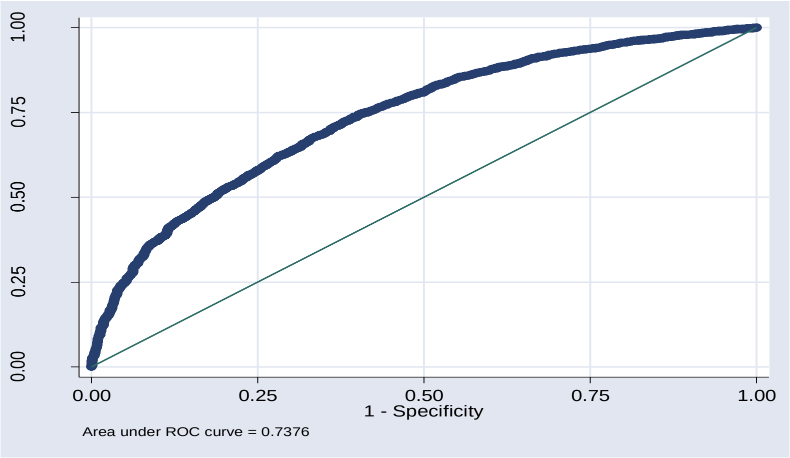

Fig 1: Receiver operating characteristics curve for anemia of included children age 6-59 months selected from the 2016 EDHS (n=7790)

Results

Individual-level characteristics of study subjects

Seven thousand seven hundred ninety (7790) children 6 -59 months of age were included in this study. Above half (52%) of the children were male, and 34.2%, 33.3% and 32.2% where in the age category of 6-23, 24-41 and 42-59 months of age, respectively with mean ± SD (standard deviation) of 32 ±15 months. The prevalence of anemia was 57.6% with a median hemoglobin concentration of 10.7 (IQR: 9.6-11.6).

Almost all of the mothers (caretakers) (95%) were living with their respective partners and most of them were Muslims (40%) followed by Orthodox Christians (34%). Nearly half of the mothers (caretakers) (48.7%) were 20–29 years of age. The proportion of no formal education among the children's mothers (caretakers) (67%) was higher than their fathers (48.5%) (Table 1). About one-fifth (23.5%) and 13.4% of respondents fall within the poorest and richest wealth index quintiles, respectively (Fig 2).

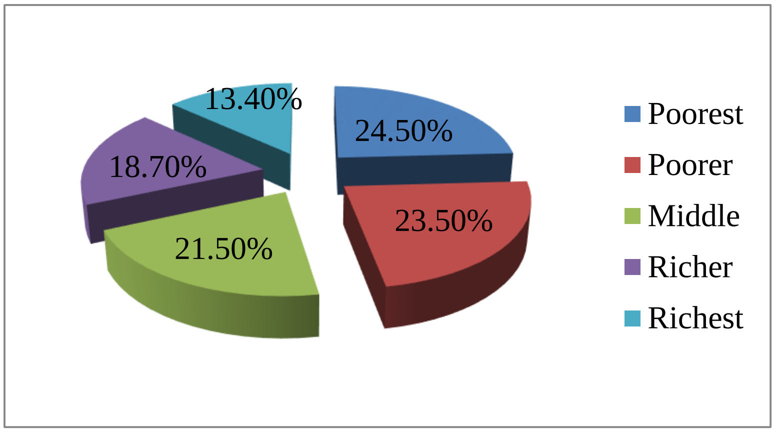

Fig 2: Characrestics of children age 6-59 months selected from the 2016 EDHS (n=7790)

Nearly half of the mothers (caretakers) (45.5%) and 92% of husbands were employed in the last twelve months prior to the survey. A quarter (25%) and more than two-fifths of children (41%) were underweight and stunted, respectively. One-third of mothers (caretakers) (30%) were anemic. Fifteen percent of children had fever two weeks prior to the survey. Above half (54.2%) of households had an unimproved type of toilet facility and about 55.5% of households had improved water sources (Table 1).

Table 1: Individual-level characteristics of included children age 6-59 months selected from the 2016 EDHS

Variable	Frequency (weighted)	Percentage (%)
**Child Sex**		
Male	4039	51.8
Female	3751	48.2
**Child age (months)**		
6-23	2667	34.2
24-41	2597	33.3
42-59	2526	32.4
**Birth weight**		
Average	3276	42.1
Smaller than average	2001	25.7
Larger than average	2513	32.3
**Birth order**		
1	1362	17.5
2-3	2417	31
4-5	1870	24
>=6	2141	27.5
**Religion**		
Orthodox	2677	34.4
Protestant	1722	22.1
Muslim	3153	40.5
Other	238	3.1
**Maternal age**		
15-19	208	2.7
20-29	3796	48.7
30-39	3065	39.3
40-49	721	9.3
**Maternal educational level**		
No education	5224	67.1
Primary	2091	26.8
Secondary	318	4.1
Higher	157	2
**Husband educational level**		
No education	3582	48.5
Primary	3008	40.7
Secondary	526	7.1
Higher	275	3.7

Table 1: Individual-level characteristics of included children age 6-59 months selected from the 2016 EDHS (*Continued*)

Variable	Frequency (weighted)	percentage
**Maternal employment status**		
No	4248	54.5
Yes	3542	45.5
**Husband employment status**		
No	599	8.1
Yes	6792	91.9
**Source of drinking water**		
Improved	4320	55.5
Unimproved	3470	44.5
**Types of toilet facility**		
Improved	719	9.2
Unimproved	4221	54.2
No facility	2850	36.6
**Child wasting**		
No	7049	90.6
Yes	731	9.4
**Child underweight**		
No	5789	74.7
Yes	1964	25.3
**Child stunting**		
No	4490.	59
Yes	3175	41
**Maternal anemia**		
No	5381	70
Yes	2320	30
**Child deworming**		
No	6787	87
Yes	1003	13
**Child diarrhea**		
No	6788	87.14
Yes	1002	12.86
**Child fever**		
No	6605	84.79
Yes	1185	15.21
**Symptoms of acute respiratory infection**		
No	6157	79
Yes	1633	21

Community-level characteristics of study subjects

About nine in ten (89.9%) of the respondents were rural area residents. Most of the respondents were from Oromia (43.9%) followed by SNNP (21.1%) and Amhara (19.5%) region. Nearly half (47.4%) of the respondents were living in communities with a high proportion of women unemployment. The above half (62.2%) of mothers were from communities with lower poverty status. Above half of mothers (52.5%) were from communities with a low proportion of women education (Table 2).

Table 2: Community-level characteristics of included children age 6-59 months selected from the 2016 EDHS

Variables	Frequency (weighted)	Percentage (%)
**Region**		
Tigray	526	6.8
Afar	76	0.98
Amhara	1520	19.5
Oromia	3418	43.9
SNNP	1637	21.1
Benishangul Gumuz	83	1.1
Gambela	18	0.23
Somali	321	4.11
Harari	15	0.2
Addis Abeba	148	1.9
Dire Dawa	29	0.37
**Place of residence**		
Rural	7002	89.9
Urban	788	10.1
**Community-women education**		
Low	4092	52.5
High	3698	47.5
**Community- women unemployment**		
Low	4098	52.6
High	3692	47.4
**Community -poverty**		
Low<br>High	4098<br>2945	62.2<br>37.8

Key: SNNP:-Sothern Nations and Peoples of Ethiopia

Distribution of anemia by individual and community level factors

Under this subtitle, the distribution of anemia by the factors which had significant association with anemia (Table 4) among children aged 6-59 months was elaborated. The highest proportion of anemia (72%) was observed in children 6-23 months old as opposed to the lowest proportion in children 42-59 months old (43%) (Fig 3).

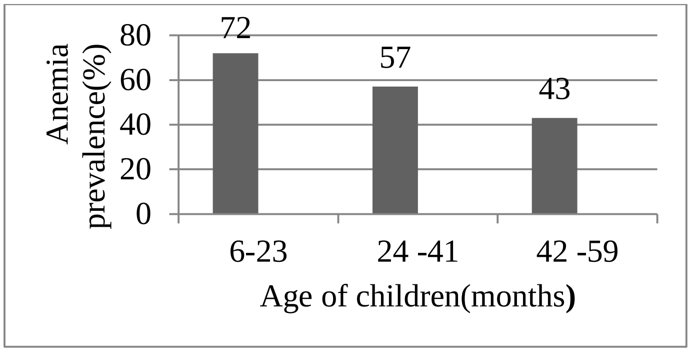

Fig 3: Anemia distribution by age of children in Ethiopia: A multilevel analysis using EDHS 2016 (n=7790)

The highest proportion of anemia among children 6-59 months of age was observed in Somali (83%) and Afar (75%) as opposed to the lowest percentage recorded in Amhara (42%) (Fig 4).

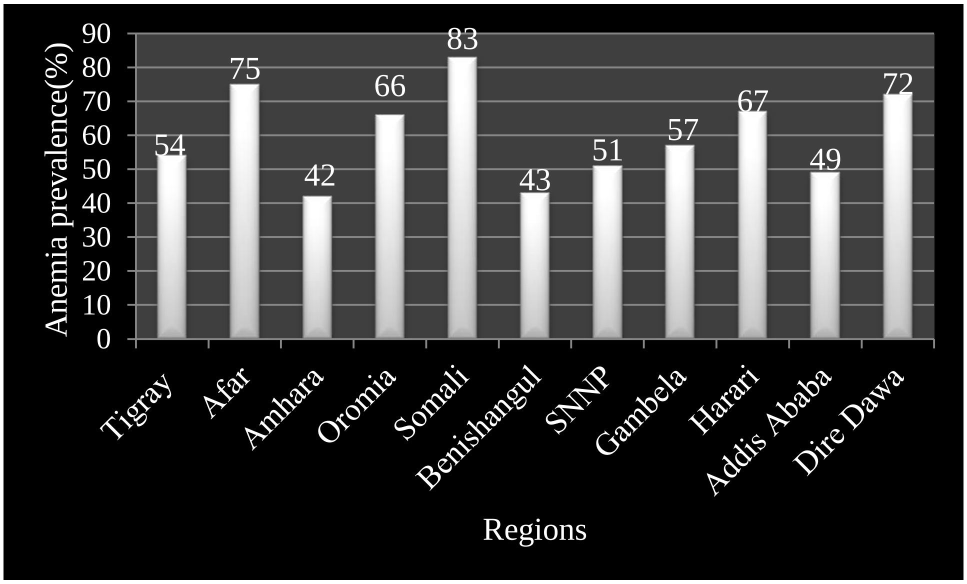

Fig 4: Percentage distribution of anemia among children 6-59 months by Region of respondents in Ethiopia: A multilevel analysis using EDHS 2016 (n= 7790).

Children from anemic mothers (69%) had higher prevalence of anemia as compared to children whose mothers (caretakers) were not anemic (Fig 5).

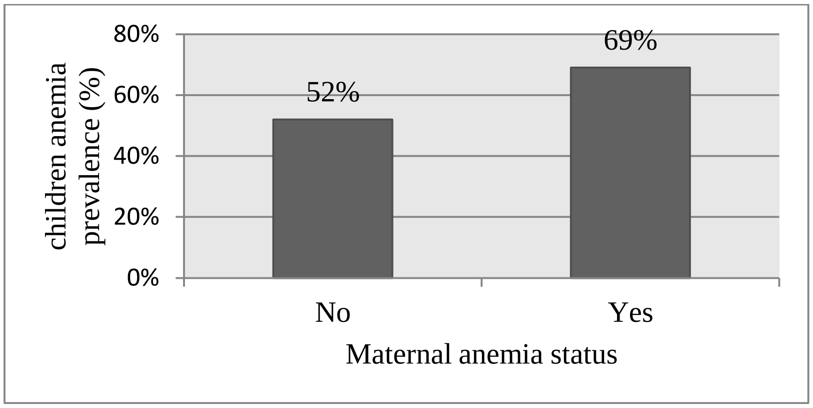

Fig 5: Distribution of anemia prevalence among children 6-59 months in Ethiopia: A multilevel analysis using EDHS 2016 (n=7790)

Individual and community-level factors associated with anemia

From the individual-level factors, anemia was most strongly associated with child age, wealth index, maternal anemia and child stunting followed by child underweight, child fever and birth order whereas from the community-level, the strongest odds of anemia occurred among children from Somali, Harari, Dire Dawa and Afar region followed by Oromia and Addis Ababa regions (Table 4).

The odds of anemia were 4.45 (AOR=4.45; 95%CI; 3.62, 5.36) and 1.83 (AOR=1.83; 95% CI: 1.59, 2.09) times higher for children 6–23 and 24-41 months of age than children at age 42–59 months, respectively. Children from the poorest families had 1.51 (AOR=1.51; 95% CI: 1.11, 2.04) times higher odds of anemia than children from the richest families. The odds of anemia were 1.26 (AOR=1.26; 95% CI: 1.00, 1.61) times higher for children with birth order six and above than first-order children (Table 4).

Underweight children had 1.34 (AOR=1.34; 95% CI: 1.14, 1.57) times higher odds of anemia than children who were not underweight. The odds of anemia were 1.40 (AOR=1.40; 95 % CI: 1.24, 1.59) times higher among children who were stunted than children who are not stunted. Maternal anemia was positively associated with childhood anemia. Children whose mothers were anemic had 1.42 (AOR=1.42; 95% CI: 1.21, 1.55) times higher odds of anemia than children from non-anemic mothers. The odds of anemia were 1.32 (AOR=1.32; 95% CI: 1.09, 1.60) times higher for children who suffered a fever two weeks prior to the survey than children with no fever (Table 4).

The region was a significant predictor of anemia among children. Children from Somali, Dire Dawa and Harari had 3.38 (AOR=3.38; 95% CI: 3.25, 5.07), 2.22 (AOR=2.22; 95% CI: 1.42, 3.48) and 1.88 (AOR=1.88; 95% CI: 1.23, 2.88) times higher odds of anemia than children, respectively than children from Tigray region. The odds of anemia were 0.71 (AOR=0.71; 95% CI: 0.52, 0.96) and 0.58 (AOR=0.58; 95% CI: 0.40, 0.82) times lower among children who were living in Amhara and Benishangul than children, respectively than children from Tigray region. Children who were living in communities of lower poverty status had 0.81 (AOR=0.81; 95% CI: 0.66, 0.99) times lower odds of anemia than others (Table 4).

Table 4: Individual and community-level factors associated with anemia among children age 6-59 months selected from the 2016 EDHS

Variables	Anemia Status Frequency (%)		AOR [95%CI]	P-value
	No	Yes		
**Child age (months)**				
6-23	746.5 (28)	1921 (72)	4.45 [3.62, 5.36]	0.000
24-41	1116 (43)	1481 (57)	1.83 [1.59, 2.09]	0.000
42-59	1444 (57)	1082 (43)	1	
**Wealth index**				
Poorest	583 (32)	1245 (68)	1.51[1.11, 2.04]	0.035
Poorer	770 (42)	1058 (58)	1.29 [0.98, 1.72]	0.052
Middle	772 (46)	901 (54)	1.11 [.84, 1.46]	0.296
Richer	639 (45)	776 (55)	1.19 [.90, 1.55]	0.106
Richest	542 (52)	504 (48)	1	
**Birth order**				
1	602 (44)	760 (55.8)	1	
2-3	1053 (43)	1365 (56.5)	1.08[.91, 1.28	0.112
4-5	779 (42)	1100 (58.4)	1.16 [.94, 1.43]	0.062
>=6	873 (40.8)	1268 (59.2)	1.26 [1.00, 1.61]	0.044
**Child underweight**				
No	2591 (45)	3198. (55)	1	
Yes	701 (36)	1262 (64)	1.34 [1.14, 1.57]	0.000
**Child stunting**				
No	2052 (46)	2438 (54)	1	
Yes	1223(39)	1953 (61)	1.4 [1.24, 1.59]	0.000
**Maternal anemia**				
No	2563 (48)	2818 (52)	1	
Yes	723 (31	1597 (69)	1.42 [1.21, 1.55]	0.000
**Child fever**				

No	2886 (44)	3719 (56)	1	
Yes	420 (35)	764 (65)	1.32[1.09, 1.60]	0.004
**Community-poverty**				
High	1020 (35)	1925 (65)	1	
Low	2287 (47)	2558 (53)	0.81[.66, .99]	0.032
**Region**				
Tigray	242 (46)	284 (54)	1	
Afar	19 (25)	57 (75)	1.66 [1.11, 2.48]	0.001
Amhara	873 (57)	647 (42)	0.71 [0.52, 0.96]	0.089
Oromia	1170 (34)	2,248 (66)	1.62 [1.16, 2.26]	0.000
Somali	54 (17)	267 (83)	3.38 [3.25, 5.07]	0.000
Benishangul	47 (57)	36 (43)	.58[.40, .82]	0.000
SNNP	804 (49)	833 (51)	1.00 [0.71, 1.42]	0.113
Gambela	8 (43)	10 (57)	1.20 [0.81, 2.78]	0.052
Harari	5 (33)	10 (67)	1.88 [1.23, 2.88]	0.000
Addis Ababa	76 (51)	72 (49)	1.54[1.01, 2.35]	0.001
Dire Dawa	8 (28)	21 (72)	2.22 [1.42, 3.48]	0.000

Key: 1- reference category, AOR- Adjusted Odds Ratio, CI- Confidence Interval, SNNPR- Southern Nations, Nationalities and People's Region

Discussion

This study aimed to identify individual and community-level factors associated with anemia among children aged 6-59 months., we found that anemia among children aged 6-59 was most strongly associated with individual-level factors such as child age, wealth index, maternal anemia and child stunting followed by child underweight, child fever and birth order, whereas from the community-level the strongest odds of anemia occurred among children from Somali, Harari, Dire Dawa and Afar region followed by Oromia and Addis Ababa. This was also supported by the observed heterogeneity in odds of anemia between communities.

Child age was negatively associated with anemia in which the odds of anemia decreased as the age of child increased. Children 6-23 months old had higher odds of anemia as compared to children 42-59 months old. This result is in line with some previous studies done in Bangladesh and Ethiopia (13, 18, 28). This might be due to children experiencing intense growth and development in the first 2 years of life, resulting in a high demand for iron (29). Additionally, complementary foods are often initially rejected by the infant, thereby exacerbating the risk of anemia.

Children with six and above birth order had higher odds of anemia than first-order children. This result is similar to findings from a prior study done in New Delhi, India (30). This could be due to increasing birth order might relate to maternal depletion of iron; as the finding of this study showed that maternal anemia leads to child anemia.

Children from the poorest households had higher odds of anemia than children from richest households. This finding is in agreement with what was reported in Bangladesh, Malawi and Ethiopia (11, 21, 28). This could be explained as poor families are less likely to afford adequate and diversified foods and access to health care which leads to poor child health outcomes.

The odds of anemia were higher for children from anemic mothers than non-anemic mothers. This is supported by studies conducted in India and Ethiopia (13, 31). The reasons may be mothers and children share common home environments, socioeconomic, and dietary conditions. Moreover, maternal iron deficiency is associated with low birth weight; even children born with adequate weight have reduced iron reserves when their mothers are anemic (32).

This study also revealed that, being underweight or stunted was positively associated with anemia. The association between these anthropometric indices and anemia has been observed in other studies (13, 15, 21, 28). The possible explanation could be stunting, underweight and anemia are all caused by malnutrition, and thus follow a similar causal pathway that is; feeding children less than four times a day and low dietary diversity. Another explanation could be nutritional inadequacies may impair immunity with a repeated infection which, in turn, depletes iron stores.

The presence of fever in the last two weeks prior to the survey was found to be a significant determinant of anemia. This finding confirms the findings from Indonesia, Burma, Nigeria and Malawi (21, 33-35). This could explain as fever is a symptom of acute febrile illness such as malaria; which might cause red blood cell destruction. Inflammation also decreases red blood cell production (36). Another explanation could be that sick children are known to have poorer appetites; hence a lower dietary intake.

This study found that parents' educational level had no significant association with anemia among children aged 6-59 months. This finding is in contrast with several other studies where an association was observed, especially with maternal educational level (18, 28, 35). The possible explanation could be controlling socio-economic factors such as parents' employment, wealth-index, and the composite factors like community poverty in the model may have a more pronounced effect in determining childhood anemia than educational level.

Region was found to be significantly associated with anemia. This finding is supported by other studies from Ghana and Ethiopia (18, 37). Children from Somali, Dire Dawa, Harari and Afar regions had higher odds of anemia than children from Tigray. This could be because of differences in living standards, socioeconomic status and cultural norms regarding feeding habits among regions. People who are living in Afar and Somalia make their living from livestock production and the main daily meal is milk. A previous study documented that milk reduces the bioavailability of iron which leads to anemia (38). Another finding of this study was the significant association of community poverty status and anemia among children. Children who live in communities with high poverty status had higher odds of anemia than others. This

significant association might be due to less access to health care, fewer job opportunities and lack of other social services within the community which resulted in lower-income.

Limitations

The limitations of this study include: not controlling variables of dietary intake and child feeding practices due to missing values. Other possible child-related explanatory variables such as parasitic infection and chronic illness were not included in the analysis because these variables are not in the EDHS data. The data based on self-reporting are limited by recall and misclassification biases, and only children living at the time of the survey were included. In addition, the community variability in the combined model was 7.03%. This indicates that there are still other variables that are not controlled. These variables could be parasitic infection, immunization history, chronic infections, dietary intake and child feeding practices.

Conclusion

We found that anemia among children aged 6-59 was most strongly associated with individual-level factors such as child age, wealth index, maternal anemia and child stunting followed by child underweight, child fever and birth order whereas from the community-level the strongest odds of anemia occurred among children from Somali, Harari, Dire Dawa and Afar region followed by Oromia and Addis Ababa region. Interventions like community-based screening for early detection and management of stunted and underweight children should strengthen to reduce anemia. As our model predicts, wealth index has additional contribution to anemia in Ethiopian children 6-59 months. This suggests that interventions targeted to the improvement of Economic subsidy may contribute to a reduction in childhood anemia and its devastating complications. In addition, special attention should be given to children less than two years of age. Similarly, priority should be given to regions such as Somali, Harari and Afar during the implementation of interventions to reduce anemia.

Acknowledgments

The authors would like to thank the Department of Public Health, Adigrat University, and Mekelle University, College of Health Sciences, Tigray, Ethiopia. The authors also acknowledge EDHS, DHS program and ICF International for providing us permissions to access the data set.

Author contributions

Conception: Menaseb Gebrehaweria

Design: Menaseb Gebrehaweria**,** Afework Mulugeta, Abate Bekele, Lire Lemma, Muzey Gebremichael, Haftay Gebremedhin, Berhe Etsay, Tesfay Tsegay, Yared Hailesilassie, Yohannes Kinfe, Fre Gebremeskel, Letemichael Mezgebo, Selam Shushay

Acquisition: Menaseb Gebrehaweria

Formal analysis: Menaseb Gebrehaweria

Interpretation: Menaseb Gebrehaweria**,** Afework Mulugeta, Abate Bekele, Lire Lemma, Muzey Gebremichael, Haftay Gebremedhin, Berhe Etsay, Tesfay Tsegay, Yared Hailesilassie, Yohannes Kinfe, Fre Gebremeskel, Letemichael Mezgebo, Selam Shushay

Drafting manuscript: Menaseb Gebrehaweria

References

1. World Health Organization. Haemoglobin concentrations for the diagnosis of anaemia and assessment of severity. Vitamin and Mineral Nutrition Information System. Geneva: World Health Organization; 2011.

2. World Health Organization. Iron deciency anemia Assessment, Prevention and Control. A guide for program managers. Geneva: World Health Organization; 2001.

3. World Health Organization. Worldwide prevalence of anemia 1993–2005. WHO Global Database on Anemia; 2008.

4. Central Statistical Agency [Ethiopia]. Ethiopia Demographic and Health Survey 2016. Addis Ababa, Ethiopia, and Rockville, Maryland; 2016.

5. World Health Organization. The global prevalence of anemia in 2011. Geneva: World Health Organization; 2015.

6. Magalhaes RJS, Clements AC. Spatial heterogeneity of haemoglobin concentration in preschool-age children in sub-Saharan Africa. Bull World Health Organ. 2011;89(6):459-68.

7. Central Statistical Agency [Ethiopia], ICF International. Ethiopia Demographic and Health Survey 2011. Addis Ababa, Ethiopia and Calverton, Maryland, USA. Central Statistical Agency and ICF International; 2012.

8. Federal Democratic Republic of Ethiopia. National Nutrition Programme June 2013-June 2015; 2015.

9. World Health Organization. Global nutrition targets 2025. anaemia policy brief (WHO/NMH/NHD/144) Geneva: World Health Organization; 2014.

10. Bernard J. Brabin ZP, Francine Verhoeff. An Analysis of Anemia and Child Mortality. The journal of Nutrition 2001;131(2):636-48.

11. Woldie H, Kebede Y, Tariku A. Factors Associated with Anemia among Children Aged 6–23 Months Attending Growth Monitoring at Tsitsika Health Center, Wag-Himra Zone, Northeast Ethiopia. Journal of Nutrition and Metabolism. 2015.

12. Jemal Y, Haidar J, Makau WK. The magnitude and determinants of anaemia among refugee preschool children from the Kebribeyah refugee camp, Somali region, Ethiopia. South African Journal of Clinical Nutrition. 2017;30(1):1-6.

13. Muchie KF. Determinants of severity level of anemia among children aged 6-59 months in Ethiopia: Further analysis of the 2011 Ethiopian Demographic and health survey. BMC Nutrition 2016;2(51).
14. Gebreegziabiher G, Etana B, Niggusie D. Determinants of Anemia among Children Aged 6–59 Months Living in Kilte Awulaelo Woreda, Northern Ethiopia. Hindawi Publishing Corporation.; 2014.
15. Gari T. Anaemia among children in a drought affected community in south-central Ethiopia. PLoS ONE. 2017;12(3).
16. Rosen G. A history of public health. Baltimore: Johns Hopkins University Press. 1993.
17. Heck RH, Thomas SL, Tabata LN. Multilevel and longitudinal modeling with IBM SPSS2010.
18. Kawo KN, Asfaw ZG, Sebro NY. Multilevel Analysis of Determinants of Anemia Prevalence among Children aged 6-59 Months in Ethiopia: Classical and Bayesian approaches. 2016.
19. Dey S, Raheem E. Multilevel multinomial logistic regression model for identifying factors associated with anemia in children 6–59 months in northeastern states of India. Cogent Mathematics. 2016.
20. Ngnie-Teta I, Receveur O, Defo BK, Receveur O. Risk factors for moderate to severe anemia among children in Benin and Mali: Insights from a multilevel analysis. Food and Nutrition Bulletin. 2007;28(1).
21. Ngwira A, Kazembe L. Analysis of severity of childhood anemia in Malawi: a Bayesian ordered categories model. Open Access Medical Statistics; 2016;6:9-20.
22. Stoltzfus JC. Logistic Regression: A Brief Primer. Academic Emergency Medicine.; 2011.
23. Ali S, Ali A, Khan SA, Hussain S. Sufficient Sample Size and Power in Multilevel Ordinal Logistic Regression Models. Computational and Mathematical Methods in Medicine; 2016.
24. Steenbergen MR. The Multilevel Logit Model for Binary Dependent Variables; 2012.
25. Hox JJ. Multilevel Analysis: Techniques and Applications second ed. Great Britain: Routledge; 2010.
26. Williams R. Multicollinearity. http://www3ndedu/william/; 2015.
27. Hosmer DW, Lemeshow S. Applied Logistic Regression. Second Edition ed; 2000.

28. Khan JR, Awan N, Misu F. Determinants of anemia among 6–59 months aged children in Bangladesh: evidence from nationally representative data. BMC Pediatrics. 2016;16(3).
29. Tengco L, Rayco-p S, Solon J, Sarol J, SAlon F. Determinants of anemia among preschool children in the philippines. J Am coll nutr 2008;27(2):229-43.
30. Ray S, Chandra J, Jayashree Bhattacharjee, Sharma S, Agarwala A. Determinants of nutritional anaemia in children less than five years age in New Delhi, India International Journal of Contemporary Pediatrics. 2016;3(2):403-8.
31. Singh RK, Patra S. Extent of Anaemia among Preschool Children in EAG States, India: A Chllenge to Policy Makers. Hindawi Publishing Corporation. 2014.
32. Allen L. Biological mechanisms that might underlie iron's effects on fetal growth and preterm birth. J Nutr. 2001;131(2):581–9.
33. Semba R, Pee Sd, Ricks M, Sari M, Bloem MW. Diarrhea and fever as risk factors for anemia among children under age five living in urban slum areas of Indonesia. International Journal of Infectious Diseases. 2007;12:62-70.
34. Gayawan E, Arogundadeb E, Adebayo S. Possible determinants and spatial patterns of anaemia among young children in Nigeria: a Bayesian semi-parametric modeling Int Health 2014;6:35–45.
35. Ai Zhao YZ, Ying Peng, Jiayin Li, Titi Yang, Zhaoyan Liu, Yanli Lv, Peiyu Wang. Prevalence of Anemia and Its Risk Factors Among Children 6–36 Months Old in Burma. 2012.
36. Quintero JP, Siqueira AM, Tobon A, Blair S, Mreno A, Herrera MA, et al. Malaria-related anemia: a Latin American perspective. Men Inst Oswaldo cruz. 2011;106:91-104.
37. Fosu MO, Frimpong FO, Arthur MO. Factors Associated with Haemoglobin Prevalence among Ghanaian Children Aged 6 – 59 months. Journal of Biology, Agriculture and Healthcare; 2014;4(2).
38. Kejo D, Petrucka PM, Martin H, Kimanya ME, Mosha TC. Prevalence and predictors of anemia among children under 5 years of age in Arusha District, Tanzania. Pediatric Health, Medicine and Therapeutics; 2018.

Supporting information

S1 File. Authorization letter.
(PDF)

S2 File. Child dataset final.
(DTA)

Annex 1: Random effects estimates of anemia for included children age 6-59 months selected from the 2016 (n=7790)

**Estimates**	**Null mode**	**Model 1**	**Model 2**	**Model 3**
Variance ($\delta^2 u_0$)	0.76*	0.36*	0.28*	0.25*
ICC (%)	18.77	10.03	8.04	7.3
PCV (%)	(ref)	52.6	63.2	67
Model comparison AIC	9967	8373.8	9692.9	8281

Key: AIC – Akaike Information Criteria, ICC - Intraclass Correlation Coefficient

PCV – Percentage Change in Variance,* p-value<0.001

DHS Datasets Authorization letter

Click here to access/download
Supporting Information
AuthLetter_117393.pdf

Child Dataset in STATA format

Click here to access/download
Supporting Information
child normalized w.dta

Child data set final

Click here to access/download
Supporting Information
child data set final.dta

Click here to access/download
Supporting Information
Authorization letter.pdf

INDIVIDUAL AND COMMUNITY LEVEL FACTORS ASSOCIATED WITH ANEMIA AMONG CHILDREN 6 - 59 MONTHS OF AGE IN ETHIOPIA: A FURTHER ANALYSIS OF 2016 ETHIOPIA DEMOGRAPHIC AND HEALTH SURVEY

Menaseb Gebrehaweria[1*], Afework Mulugeta[2], Abate Bekele[3], Lire Lemma[4], Muzey Gebremichael[1], Haftay Gebremedhin[1], Berhe Etsay[1], Tesfay Tsegay[5], Yared Haileslasie[5], Yohannes Kinfe[6], Fre Gebremeskel[1], Letemichael Mezgebo[1], Selam Shushay[7]

1 Department of Public Health, College of Medicine and Health Sciences, Adigrat University, Tigray, Ethiopia
2 Department of Nutrition, School of Public Health, College of Health Sciences, Mekelle University, Tigray, Ethiopia,
3 Department of Biostatistics, School of Public health, College of Health Sciences, Mekelle University, Tigray, Ethiopia
4 Department of Public Health, College of Medicine and Health Sciences, Wachamo University, Sothern Nations Nationalities and Peoples of Ethiopia
5 Department of Nursing, College of Medicine and Health Sciences, Adigrat University, Tigray, Ethiopia
6 Department of Public Health, College of Medicine and Health Sciences, Aksum University, Tigray, Ethiopia
7 Department of Midwifery, College of Medicine and Health Sciences, Adigrat University, Tigray, Ethiopia
[1*] Email-menasebgh@gmail.com

Abstract

Background: Anemia is a global public health problem; but its burden is disproportionately borne among children in the African Regions. The 2016 Ethiopia Demographic and Health Survey report showed that the prevalence of anemia among children 6-59 months of age was 57%; far exceeding the national target of 25% set for 2015. Although studies have been conducted in Ethiopia, multilevel analysis has rarely been used to identify factors associated with anemia among children. Therefore, this study ~~was~~ aimed to identify individual and community-level factors associated with anemia among children 6-59 months of age by fitting a multilevel logistic regression model.

Methods: The data was obtained from the 2016 Ethiopia Demographic and Health Survey, conducted from January to June 2016, and downloaded from the website http://www.DHSprogram.com. The sample was taken using two-stage stratified sampling. In stage one, 645 Enumeration Areas and in stage two 28 households per Enumeration Area were selected. A sample of 7790 children 6-59 months of age was included. Data were analyzed using STATA version 14. A multilevel logistic regression model was fitted and an adjusted odds ratio with a 95% confidence interval was obtained.

Result: From the individual-level factors, anemia was associated most strongly with child age, wealth index, maternal anemia and child stunting followed by child underweight, child fever and birth order whereas from the community-level, the strongest odds of anemia occurred among children from Somali, Harari, Dire Dawa and Afar region followed by Oromia and Addis Ababa. Low community-poverty is a protective factor for anemia. The odds of anemia were 0.81 (AOR=0.81; 95% CI: 0.66, 0.99) times lower for children who were living in communities of lower poverty status than children who were living in communities of higher poverty status. Children from Somali and Dire Dawa had 3.38 (AOR=3.38; 95% CI: 3.25, 5.07) and 2.22 (AOR=2.22; 95% CI: 1.42, 3.48) times higher odds of anemia, respectively than children from the Tigray region.

Conclusions: This study ~~showed~~ shows that anemia among children 6-59 months of age is affected both by the individual and community level factors. It is better to strengthen the strategies of early detection and management of ~~malnutrition~~ stunted and underweight children.

At the same time, interventions should be strengthened to address maternal anemia, child fever and poverty, specifically targeting regions identified to have a high risk of anemia.

Introduction

Globally, anemia affects around 800 million children and women; of which 42.6% are children (1). African countries have the highest prevalence (62.3%) followed by South-East Asia (53.8%) and Eastern Mediterranean Region (48.6%) (1). Furthermore, it is a major public health problem in Sub-Saharan African countries with much national prevalence estimated to be above 40% (2).

According to the 2011 Ethiopia Demographic and Health Survey (EDHS), anemia prevalence among children 6-59 months of age was 44% (3). In addition, the 2016 EDHS report showed that prevalence of anemia among children 6-59 months of age was 57% (4). This indicates that anemia is a severe public health problem which increased by 13% within five years period, during a period when the government of Ethiopia undertook efforts such as vitamin ‘A’ supplementation, deworming, and use of fortified foods to reduce anemia through national nutrition programs (5).

Anemia is an important indicator of poor nutrition and health with major consequences of socioeconomic development (6). Children younger than two years of age with severe anemia are at increased risk of mortality and, even mild forms, which might be corrected cause permanent cognitive damage by decreasing attention span and shortening of memory (7).

Although biochemical and hematological tests exist, hemoglobin concentration in the blood is the most reliable indicator of anemia at the population level (8). This latter method was used to assess for anemia among children aged 6-59 months in the 2016 Ethiopia Demographic Health Survey (EDHS) (4).

According to the WHO criteria, anemia in children 6-59 months of age is defined as hemoglobin concentration in the blood ~~bellow~~ below 11g/dl (9). Anemia is said to be a severe public health problem when its prevalence is 40% or more, a moderate public health problem when its prevalence is between 20 and 40% and a mild public health problem when its prevalence is between 5 and 20% in any group (10).

In Ethiopia, previous studies have linked anemia to factors such as child age, child nutritional status, parents' educational level, and wealth index (11-15). However, almost all studies used single-level analysis techniques with population groups localized in a specific study area (11, 12, 14, 15). The Single-level analysis assumes that there is no community effect beyond the characteristics of individuals (16). That is, the impact of community-level factors on anemia among children aged 6-59 months remains under-studied. Moreover, analyzing hierarchical data like the DHS using single-level analysis leads to incorrect estimation of parameters and standard errors (17).

Using multilevel analysis technique, community-level effects can be identified from individual-level effects (18-21). However, this approach has rarely been used in Ethiopia to identify factors associated with anemia among children. One study that used multilevel-analysis technique failed to examine the effect of some factors such as poor nutritional status of children (stunting, wasting and underweight), child health-related factors (fever, diarrhea and respiratory infection), maternal anemia, and variables aggregated at the community level (18).

Finally, the aims of this study were:

1. To identify individual level factors associated with anemia among children 6-59 months of age in Ethiopia.
2. To identify community level factors associated with anemia among children 6-59 months of age in Ethiopia.

Methods and Materials

Data source

Data were extracted from the nationally representative 2016 EDHS. The 2016 EDHS is the fourth survey which is implemented by the Central Statistical Agency (CSA) in collaboration with the Ethiopian Ministry of Health under the technical assistance of International Classification of Functioning, Disability, and Health (ICF) ~~thorough~~ through the DHS Program. The data were downloaded from the DHS program website, http//www.DHSprogram.com. after obtaining permission. ~~To register and get permission to use the EDHS data, the title and concept note of the thesis proposal was sent through the DHS website. Then an authorization letter was obtained from ICF international permission to use the EDHS data.~~ The EDHS data has no individual identifiers which could affect the confidentiality of participants. Accessed data were used for this study only and the EDHS data-set were not shared with the third person without direct registration.

Study population: children 6-59 months of age who were living in selected enumeration areas.

Inclusion and exclusion criteria

- ✓ Inclusion criteria: children 6-59 months of age who live in the selected enumeration areas (community).
- ✓ Exclusion criteria: children 6-59 months of age who have no hemoglobin test result.

Study design and sample size

A population-based cross-sectional survey was used to collect the 2016 EDHS data. The 2016 EDHS ~~was~~ had used a stratified two-stage cluster sampling design. Stratification was achieved by separating each region into urban and rural areas, yielding 21 sampling strata. In the first stage, 645 Enumeration Areas (EAs) or clusters were selected. Among the selected 645 EAs, 202 were in urban and 443 in rural areas. In the second stage, households were the sampling units and a fixed number of 28 households per each EA were selected. From the total of 10,641 under-five years old children, 9504 were children 6-59 months of age. Data on hemoglobin level from the survey were available for 7790 children.

Definitions of s~~S~~tudy variables

We assessed the impact of individual and community-level variables on anemia among children 6-59 months of age. We defined anemia in 6-59 month age children as hemoglobin <11 g/dL according to WHO criteria (8). Individual level variables were: sex, age, birth order, birth weight, religion, number of under-five children, childhood wasting, underweight, stunting, symptoms of acute respiratory infection, child fever and diarrhea, maternal anemia and age, parents' educational and employment status, wealth index, source of drinking water, and type of toilet facility, whereas community-level variables were: region, community-poverty, community-women education and community- women unemployment.

Wealth index~~:~~ is a composite measure of a household's cumulative living standard. It was calculated based on household ownership of selected assets such as televisions and bicycles, cars; materials used for the housing construction; source of drinking water; and type of sanitation facilities. It was then generated using principal components analysis and the individual households were placed on a continuous scale of relative wealth. In the EDHS all mothers and children were assigned a standardized wealth index score. It was measured as a composite variable made up of five quintiles as poorest, poorer, middle, richer and richest (4).

Anthropometrics: stunting was defined as height or length for age (HFA) <-2SD (standard deviation), wasting as weight-for-height (WFH) <-2 SD and underweight as weight-for-age (WFA) <-2 SD.

The community-level variables that directly measure the community characteristics in the 2016 EDHS were the place of residence (rural or urban) and region (either of the nine regions or the two administrative cities). We created also other additional variables by aggregating the individual level's characteristics within their respective clusters. These variables were: community poverty, community women education and community women unemployment.

Community-poverty~~:~~ is the proportion of mothers who reside in poor or poorest households in the community. The aggregate of the poorest or poor individuals can show the overall poverty of the cluster. For this proportion, the median value was calculated as summary statistics and categorized as 'High' or 'Low' based on this national median value.

Community-women education (CWE): is Pproportion of mothers aged 15–49 with secondary or higher education in the community. The median value was calculated as summary statistics that represent the educational status of women in the cluster and was categorized as ‘High’ or ‘Low’ based on the national median value.

Community-women unemployment status (CWUe): is Pproportion of mothers aged 15–49 who were not employed in the community in the past twelve months. It was categorized as high if clusters had more than or equal to the national median proportion of unemployed mothers or low otherwise.

Methods of data analysis

Before doing any analysis, weighting and normalization were done for the sample in order to ensure the representativeness of the sample to different regions and their place of residence. Data were analyzed by Stata version 13 and a multilevel binary logistic regression model was fitted. Frequencies, percentages, graphs and charts were used to describe categorical variables. The effect of each predictor variable on the outcome variable was checked at a significance level of $p \leq 0.25$ independently (22). Variables that are statistically significant at the bivariate multilevel logistic regression analysis were considered as candidates for multivariable analysis. Accordingly, in the multivariable analysis the following variables were adjusted and controlled: number of <5 children in the household, child age, religion of mother (caretaker), birth order, parents employment status and educational level, maternal age, type of toilet facility, source of drinking water, wealth index, child stunting, wasting, underweight, fever, diarrhea, child deworming, symptoms of acute respiratory infection, maternal anemia, community- women education, place of residence, community-poverty, region and community- women unemployment. Adjusted Odds Ratio (AOR) with 95% Confidence Interval (CI) at a significance level of $p<0.05$ was estimated. The result of multivariable analysis for individual and community-level factors associated with anemia among children aged 6-59 months is shown in Table 4.

Assuming varying intercepts across communities (clusters) but fixed coefficients, four models were developed. The first one was the null model which is fitted without independent variables; this was used to determine the variance in anemia status between the clusters in the sample.

Whereas, model one was adjusted for individual-level factors and used to examine their contribution to the variation of anemia status. Model two was adjusted for community level factors and was used for examining whether the community-level variables explain between-cluster variation on childhood anemia. Model three was developed by combining both the individual and community level variables.

Null model: for individual *i* in community j, the model can be represented as**:**

$$Y_{ij} = \Upsilon_{00} + u_{0j} + \varepsilon_{ij} \text{ ………… null model (17) (23)}$$

Where:

Y_{ij} is anemia status of ith child in the jth cluster

Υ_{00} = is the intercept; that is the probability of having anemia in the absence of explanatory variables

u_{0j} = community-level effect; ε_{ij} error at individual level

Mixed model

This model was derived by mixing both individual and community level factors simultaneously.

$$Y_{ij} = \Upsilon_{00} + \Upsilon_{k0}X_{kij} + \Upsilon_{0p}z_{pj} + u_{0j} + \varepsilon_{ij} \text{ ……(24)}$$

Where: The term γ_{k0} is the regression coefficient of the individual-level variable X_k and γ_{0p} is the regression coefficient of the community-level variable Zp. X_k and Zp were individual and community-level explanatory variables respectively. The subscripts *i* and *j* represent for the individual level and cluster number respectively.

Intraclass correlation coefficient (ICC): a measure of within-cluster homogeneity and the proportion of variance due to between-cluster differences.

$$ICC = \frac{\delta^2 u_0}{\delta^2 u_0 + \pi^2/3}$$

Where: $\delta^2{}_{u_0}$ = between cluster (community) variances and $\frac{\pi^2}{3}$ = with in cluster (community) variance. The value of $\frac{\pi^2}{3}$ in case of standard logistic distribution is 3.29 (25) .

The null model showed that there was a significant variation in anemia status among clusters (δ^2_{u0}=0.76, p-value<0.001). The ICC was 18.77%, meaning that 18.77% of the total variability in odds of anemia was due to between community differences or attributable to the unobserved factors either at community-level or at individual-level. This indicates that using a multilevel logistic regression model is better for getting valid estimates than single-level logistic regression. The variance which was due to the clustering effect decreased from 18.77% in the null model to 10.03%, 8.04% , and 7.03% in model one, model two and model three, respectively (Annex 1 ~~Table 1~~).

Proportional change in variance (PCV): calculated with reference to the null model to see relative contribution of factors to explain variation in childhood anemia.

PCV= ($\frac{V0-V1}{V0}$)*100 Where: V_0 is between community variance in the null model; V_1 is between community variance in the consecutive models (17).

Model three had the highest PCV which is 67%. This shows that 67% of the variance in the anemia status among children was due to the simultaneous effect of both individual and community-level factors found in the model (Annex 1 ~~Table 1~~).

Model diagnostics and adequacy checking

Multicollinearity was checked by using a variation inflation factor (VIF) with a cut-off point of 10 (26). It was checked for the independent variables in the final model and the VIF was found to range from 1.2 to 4.2 with mean VIF of 2.1. This shows that multicollinearity might not be a problem. Interaction effect was examined by fitting regression models that contained cross-product terms that yield no significant (p>0.05) interaction effect. Model selection was carried out by using Akaike information criteria (AIC). AIC values for each model were compared and the model with the lowest value of AIC was considered as a better explanatory model (25). Accordingly, model 4 with AIC value of 8281 was selected as the best model for explaining anemia status among children aged 6-59 months in Ethiopia (Table 1). Model accuracy was checked by the area under the curve. The Receiver Operating Characteristics curve (ROC-curve) provides a measure of the model's ability to discriminate between those subjects who experience the outcome of interest versus those who do not (27). The area under the ROC of this model was 0.7376; which means the ROC curve accuracy for outcome variable (anemia) was 74% (Fig 1).

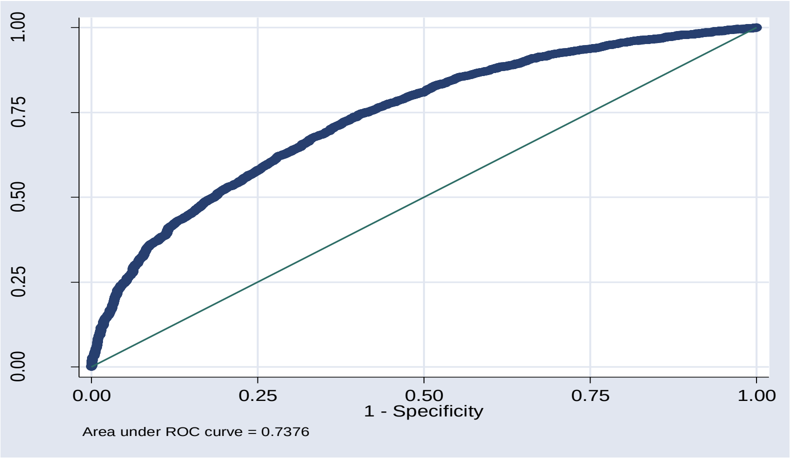

Fig 1: Receiver operating characteristics curve for anemia of included children age 6-59 months selected from the 2016 EDHS (n=7790)

Results

Individual-level characteristics of study subjects

Seven thousand seven hundred ninety (7790) children 6 -59 months of age were included in this study. Above half (52%) of the children were male, and 34.2%, 33.3% and 32.2% where in the age category of 6-23, 24-41 and 42-59 months of age, respectively with mean ± SD (standard deviation) of 32 ±15 months. The prevalence of anemia was 57.6% with a median hemoglobin concentration of 10.7 (IQR: 9.6-11.6).

Almost all of the ~~respondents~~ mothers (caretakers) (95%) were living with their respective partners and most of them were Muslims (40%) followed by Orthodox Christians (34%). Nearly half of the mothers (caretakers) (48.7%) were 20–29 years of age. The proportion of no formal education among the children's mothers (caretakers) (67%) was higher than their fathers (48.5%) (Table 1). About one-fifth (23.5%) and 13.4% of respondents fall within the poorest and richest wealth index quintiles, respectively (Fig 2).

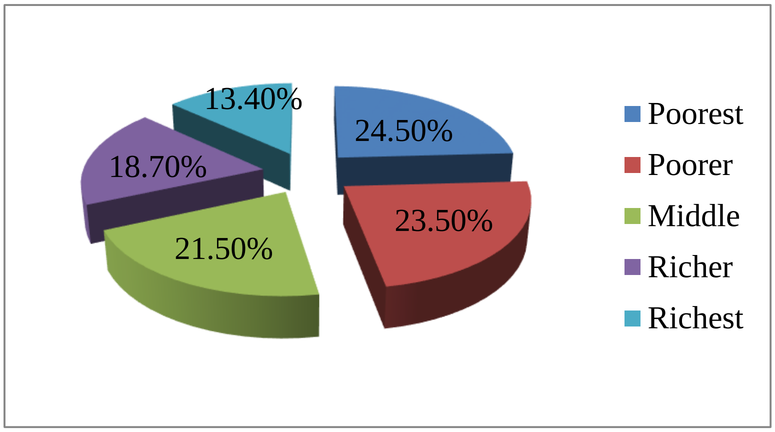

Fig 2: Characrestics of children age 6-59 months selected from the 2016 EDHS (n=7790)

Nearly half of the mothers (caretakers) (45.5%) and 92% of husbands were employed in the last twelve months prior to the survey. A quarter (25%) and more than two-fifths of children (41%) were underweight and stunted, respectively. One-third of mothers (caretakers) (30%) were anemic. Fifteen percent of children had fever two weeks prior to the survey. Above half (54.2%) of households had an unimproved type of toilet facility and about 55.5% of households had improved water sources (Table 1).

Table 1: Individual-level characteristics of included children age 6-59 months selected from the 2016 EDHS

Variable	Frequency (weighted)	Percentage (%)
**Child Sex**		
Male	4039	51.8
Female	3751	48.2
**Child age (months)**		
6-23	2667	34.2
24-41	2597	33.3
42-59	2526	32.4
**Birth weight**		
Average	3276	42.1
Smaller than average	2001	25.7
Larger than average	2513	32.3
**Birth order**		
1	1362	17.5
2-3	2417	31
4-5	1870	24
>=6	2141	27.5
**Religion**		
Orthodox	2677	34.4
Protestant	1722	22.1
Muslim	3153	40.5
Other	238	3.1
**Maternal age**		
15-19	208	2.7
20-29	3796	48.7
30-39	3065	39.3
40-49	721	9.3
**Maternal educational level**		
No education	5224	67.1
Primary	2091	26.8
Secondary	318	4.1
Higher	157	2
**Husband educational level**		
No education	3582	48.5
Primary	3008	40.7
Secondary	526	7.1
Higher	275	3.7

Table 1: Individual-level characteristics of included children age 6-59 months selected from the 2016 EDHS (*Continued*)

Variable	Frequency (weighted)	percentage
**Maternal employment status**		
No	4248	54.5
Yes	3542	45.5
**Husband employment status**		
No	599	8.1
Yes	6792	91.9
**Source of drinking water**		
Improved	4320	55.5
Unimproved	3470	44.5
**Types of toilet facility**		
Improved	719	9.2
Unimproved	4221	54.2
No facility	2850	36.6
**Child wasting**		
No	7049	90.6
Yes	731	9.4
**Child underweight**		
No	5789	74.7
Yes	1964	25.3
**Child stunting**		
No	4490.	59
Yes	3175	41
**Maternal anemia**		
No	5381	70
Yes	2320	30
**Child deworming**		
No	6787	87
Yes	1003	13
**Child diarrhea**		
No	6788	87.14
Yes	1002	12.86
**Child fever**		
No	6605	84.79
Yes	1185	15.21
**Symptoms of acute respiratory infection**		
No	6157	79
Yes	1633	21

Community-level characteristics of study subjects

About nine in ten (89.9%) of the respondents were rural area residents. Most of the respondents were from Oromia (43.9%) followed by SNNP (21.1%) and Amhara (19.5%) region. Nearly half (47.4%) of the respondents were living in communities with a high proportion of women unemployment. The above half (62.2%) of mothers were from communities with lower poverty status. Above half of mothers (52.5%) were from communities with a low proportion of women education (Table 2).

Table 2: Community-level characteristics of included children age 6-59 months selected from the 2016 EDHS

Variables	Frequency (weighted)	Percentage (%)
**Region**		
Tigray	526	6.8
Afar	76	0.98
Amhara	1520	19.5
Oromia	3418	43.9
SNNP	1637	21.1
Benishangul Gumuz	83	1.1
Gambela	18	0.23
Somali	321	4.11
Harari	15	0.2
Addis Abeba	148	1.9
Dire Dawa	29	0.37
**Place of residence**		
Rural	7002	89.9
Urban	788	10.1
**Community-women education**		
Low	4092	52.5
High	3698	47.5
**Community- women unemployment**		
Low	4098	52.6
High	3692	47.4
**Community -poverty**		
Low	4098	62.2
High	2945	37.8

Key: SNNP:-Sothern Nations and Peoples of Ethiopia

Distribution of anemia by individual and community level factors

Under this subtitle, the distribution of anemia by the some factors which had significant association with anemia (Table 4) among children aged 6-59 months was elaborated. The highest proportion of anemia (72%) was observed in children 6-23 months old as opposed to the lowest proportion in children 42-59 months old (43%).

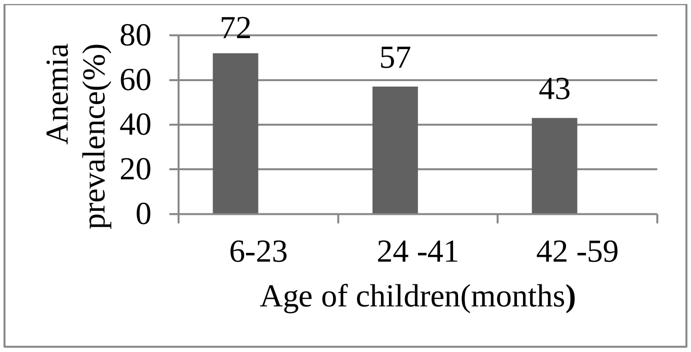

Fig 3: Anemia distribution by age of children in Ethiopia: A multilevel analysis using EDHS 2016 (n=7790)

The highest proportion of anemia among children 6-59 months of age was observed in Somali (83%) and Afar (75%) as opposed to the lowest percentage recorded in Amhara (42%).

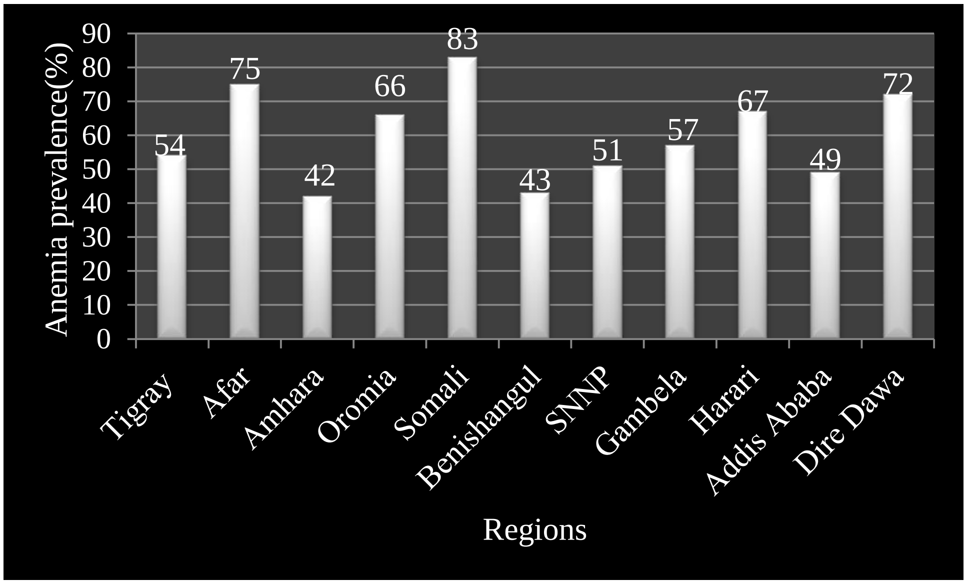

Fig 4: Percentage distribution of anemia among children 6-59 months by Region of respondents in Ethiopia: A multilevel analysis using EDHS 2016 (n= 7790).

Children from anemic mothers (69%) had higher prevalence of anemia as compared to children whose mothers (caretakers) were not anemic.

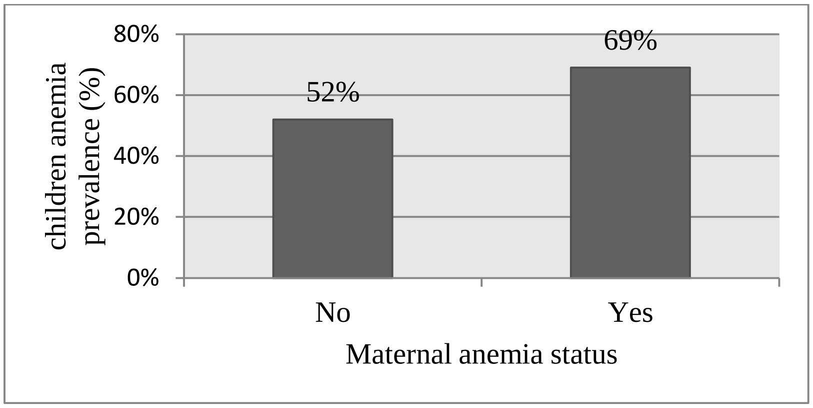

Fig 5: Distribution of anemia prevalence among children 6-59 months in Ethiopia: A multilevel analysis using EDHS 2016 (n=7790)

Individual and community-level factors associated with anemia

From the individual-level factors, anemia was most strongly associated with child age, wealth index, maternal anemia and child stunting followed by child underweight, child fever and birth order whereas from the community-level, the strongest odds of anemia occurred among children from Somali, Harari, Dire Dawa and Afar region followed by Oromia and Addis Ababa regions (Table 4).

The odds of anemia were 4.45 (AOR=4.45; 95%CI; 3.62, 5.36) and 1.83 (AOR=1.83; 95% CI: 1.59, 2.09) times higher for children 6–23 and 24-41 months of age than children at age 42–59 months, respectively. Children from the poorest families had 1.51 (AOR=1.51; 95% CI: 1.11, 2.04) times higher odds of anemia than children from the richest families. The odds of anemia were 1.26 (AOR=1.26; 95% CI: 1.00, 1.61) times higher for children with birth order six and above than first-order children (Table 4).

Underweight children had 1.34 (AOR=1.34; 95% CI: 1.14, 1.57) times higher odds of anemia than children who were not underweight. The odds of anemia were 1.40 (AOR=1.40; 95 % CI: 1.24, 1.59) times higher among children who were stunted than children who are not stunted. Maternal anemia was positively associated with childhood anemia. Children whose mothers were anemic had 1.42 (AOR=1.42; 95% CI: 1.21, 1.55) times higher odds of anemia than children from non-anemic mothers. The odds of anemia were 1.32 (AOR=1.32; 95% CI: 1.09, 1.60) times higher for children who suffered a fever two weeks prior to the survey than children with no fever (Table 4).

The region was a significant predictor of anemia among children. Children from Somali, Dire Dawa and Harari had 3.38 (AOR=3.38; 95% CI: 3.25, 5.07), 2.22 (AOR=2.22; 95% CI: 1.42, 3.48) and 1.88 (AOR=1.88; 95% CI: 1.23, 2.88) times higher odds of anemia than children, respectively than children from Tigray region. The odds of anemia were 0.71 (AOR=0.71; 95% CI: 0.52, 0.96) and 0.58 (AOR=0.58; 95% CI: 0.40, 0.82) times lower among children who were living in Amhara and Benishangul than children, respectively than children from Tigray region. Children who were living in communities of lower poverty status had 0.81 (AOR=0.81; 95% CI: 0.66, 0.99) times lower odds of anemia than others (Table 4).

Table 4: Individual and community-level factors associated with anemia among children age 6-59 months selected from the 2016 EDHS

Variables	Anemia Status Frequency (%)		AOR [95%CI]	P-value
	No	Yes		
**Child age (months)**				
6-23	746.5 (28)	1921 (72)	4.45 [3.62, 5.36]	0.000
24-41	1116 (43)	1481 (57)	1.83 [1.59, 2.09]	0.000
42-59	1444 (57)	1082 (43)	1	
**Wealth index**				
Poorest	583 (32)	1245 (68)	1.51[1.11, 2.04]	0.035
Poorer	770 (42)	1058 (58)	1.29 [0.98, 1.72]	0.052
Middle	772 (46)	901 (54)	1.11 [.84, 1.46]	0.296
Richer	639 (45)	776 (55)	1.19 [.90, 1.55]	0.106
Richest	542 (52)	504 (48)	1	
**Birth order**				
1	602 (44)	760 (55.8)	1	
2-3	1053 (43)	1365 (56.5)	1.08[.91, 1.28	0.112
4-5	779 (42)	1100 (58.4)	1.16 [.94, 1.43]	0.062
>=6	873 (40.8)	1268 (59.2)	1.26 [1.00, 1.61]	0.044
**Child underweight**				
No	2591 (45)	3198. (55)	1	
Yes	701 (36)	1262 (64)	1.34 [1.14, 1.57]	0.000
**Child stunting**				
No	2052 (46)	2438 (54)	1	
Yes	1223(39)	1953 (61)	1.4 [1.24, 1.59]	0.000
**Maternal anemia**				
No	2563 (48)	2818 (52)	1	
Yes	723 (31	1597 (69)	1.42 [1.21, 1.55]	0.000
**Child fever**				

No	2886 (44)	3719 (56)	1	
Yes	420 (35)	764 (65)	1.32[1.09, 1.60]	0.004
**Community-poverty**				
High	1020 (35)	1925 (65)	1	
Low	2287 (47)	2558 (53)	0.81[.66, .99]	0.032
**Region**				
Tigray	242 (46)	284 (54)	1	
Afar	19 (25)	57 (75)	1.66 [1.11, 2.48]	0.001
Amhara	873 (57)	647 (42)	0.71 [0.52, 0.96]	0.089
Oromia	1170 (34)	2,248 (66)	1.62 [1.16, 2.26]	0.000
Somali	54 (17)	267 (83)	3.38 [3.25, 5.07]	0.000
Benishangul	47 (57)	36 (43)	.58[.40, .82]	0.000
SNNP	804 (49)	833 (51)	1.00 [0.71, 1.42]	0.113
Gambela	8 (43)	10 (57)	1.20 [0.81, 2.78]	0.052
Harari	5 (33)	10 (67)	1.88 [1.23, 2.88]	0.000
Addis Ababa	76 (51)	72 (49)	1.54[1.01, 2.35]	0.001
Dire Dawa	8 (28)	21 (72)	2.22 [1.42, 3.48]	0.000

Key: 1- reference category, AOR- Adjusted Odds Ratio, CI- Confidence Interval, SNNPR- Southern Nations, Nationalities and People’s Region

Discussion

This study ~~was~~ aimed to identify individual and community-level factors associated with anemia among children aged 6-59 months. ~~This finding indicated that~~, we found that anemia among children aged 6-59 was most strongly associated with individual-level factors such as child age, wealth index, maternal anemia and child stunting followed by child underweight, child fever and birth order, whereas from the community-level the strongest odds of anemia occurred among children from Somali, Harari, Dire Dawa and Afar region followed by Oromia and Addis Ababa. This was also supported by the observed heterogeneity in odds of anemia between communities.

Child age was negatively associated with anemia in which the odds of anemia decreased as the age of child increased. Children 6-23 months old had higher odds of anemia as compared to children 42-59 months old. This result is in line with some previous studies done in Bangladesh and Ethiopia (13, 18, 28). This might be due to children experiencing intense growth and development in the first 2 years of life, resulting in a high demand for iron (29). Additionally, complementary foods are often initially rejected by the infant, thereby exacerbating the risk of anemia.

Children with six and above birth order had higher odds of anemia than first-order children. This result is similar to findings from a prior study done in New Delhi, India (30). This could be due to increasing birth order might relate to maternal depletion of iron; as the finding of this study showed that maternal anemia leads to child anemia.

Children from the poorest households had higher odds of anemia than children from richest households. This finding is in agreement with what was reported in Bangladesh, Malawi and Ethiopia (11, 21, 28). This could be explained as poor families are less likely to afford adequate and diversified foods and access to health care which leads to poor child health outcomes.

The odds of anemia were higher for children from anemic mothers than non-anemic mothers. This is supported by studies conducted in India and Ethiopia (13, 31). The reasons may be mothers and children share common home environments, socioeconomic, and dietary conditions. Moreover, maternal iron deficiency is associated with low birth weight; even children born with adequate weight have reduced iron reserves when their mothers are anemic (32).

This study also revealed that, being underweight or stunted was positively associated with anemia. The association between these anthropometric indices and anemia has been observed in other studies (13, 15, 21, 28). The possible explanation could be stunting, underweight and anemia are all caused by malnutrition, and thus follow a similar causal pathway that is; feeding children less than four times a day and low dietary diversity. Another explanation could be nutritional inadequacies may impair immunity with a repeated infection which, in turn, depletes iron stores.

The presence of fever in the last two weeks prior to the survey was found to be a significant determinant of anemia. This finding confirms the findings from Indonesia, Burma, Nigeria and Malawi (21, 33-35). This could explain as fever is a symptom of acute febrile illness such as malaria; which might cause red blood cell destruction. Inflammation also decreases red blood cell production (36). Another explanation could be that sick children are known to have poorer appetites; hence a lower dietary intake.

This study found that parents' educational level had no significant association with anemia among children aged 6-59 months. This finding is in contrast with several other studies where an association was observed, especially with maternal educational level (18, 28, 35). The possible explanation could be controlling socio-economic factors such as parents' employment, wealth-index, and the composite factors like community poverty in the model may have a more pronounced effect in determining childhood anemia than educational level.

Region was found to be significantly associated with anemia. This finding is supported by other studies from Ghana and Ethiopia (18, 37). Children from Somali, Dire Dawa, Harari and Afar regions had higher odds of anemia than children from Tigray. This could be because of differences in living standards, socioeconomic status and cultural norms regarding feeding habits among regions. People who are living in Afar and Somalia make their living from livestock production and the main daily meal is milk. A previous study documented that milk reduces the bioavailability of iron which leads to anemia (38). Another finding of this study was the significant association of community poverty status and anemia among children. Children who live in communities with high poverty status had higher odds of anemia than others. This

significant association might be due to less access to health care, fewer job opportunities and lack of other social services within the community which resulted in lower-income.

Limitations

The limitations of this study include: not controlling variables of dietary intake and child feeding practices due to missing values. Other possible child-related explanatory variables such as parasitic infection and chronic illness were not included in the analysis because these variables are not in the EDHS data. The data based on self-reporting are limited by recall and misclassification biases, and only children living at the time of the survey were included. In addition, the community variability in the combined model was 7.03%. This indicates that there are still other variables that are not controlled. These variables could be parasitic infection, immunization history, chronic infections, dietary intake and child feeding practices.

Conclusion

We found that anemia among children aged 6-59 was most strongly associated with individual-level factors such as child age, wealth index, maternal anemia and child stunting followed by child underweight, child fever and birth order whereas from the community-level the strongest odds of anemia occurred among children from Somali, Harari, Dire Dawa and Afar region followed by Oromia and Addis Ababa region. Interventions like community-based screening for early detection and management of stunted and underweight children should strengthen to reduce anemia. As our model predicts, wealth index has additional contribution to anemia in Ethiopian children 6-59 months. This suggests that interventions targeted to the improvement of Economic subsidy may contribute to a reduction in childhood anemia and its devastating complications. In addition, special attention should be given to children less than two years of age. Similarly, priority should be given to regions such as Somali, Harari and Afar during the implementation of interventions to reduce anemia.

Acknowledgments

The authors would like to thank the Department of Public Health, Adigrat University, and Mekelle University, College of Health Sciences, Tigray, Ethiopia. The authors also acknowledge EDHS, DHS program and ICF International for providing us permissions to access the data set.

Author contributions

Conception: Menaseb Gebrehaweria

Design: Menaseb Gebrehaweria**,** Afework Mulugeta, Abate Bekele, Lire Lemma, Muzey Gebremichael, Haftay Gebremedhin, Berhe Etsay, Tesfay Tsegay, Yared Hailesilassie, Yohannes Kinfe, Fre Gebremeskel, Letemichael Mezgebo, Selam Shushay

Acquisition: Menaseb Gebrehaweria

Formal analysis: Menaseb Gebrehaweria

Interpretation: Menaseb Gebrehaweria**,** Afework Mulugeta, Abate Bekele, Lire Lemma, Muzey Gebremichael, Haftay Gebremedhin, Berhe Etsay, Tesfay Tsegay, Yared Hailesilassie, Yohannes Kinfe, Fre Gebremeskel, Letemichael Mezgebo, Selam Shushay

Drafting manuscript: Menaseb Gebrehaweria

References

1. World Health Organization. Haemoglobin concentrations for the diagnosis of anaemia and assessment of severity. Vitamin and Mineral Nutrition Information System. Geneva: World Health Organization; 2011.

2. World Health Organization. Iron deciency anemia Assessment, Prevention and Control. A guide for program managers. Geneva: World Health Organization; 2001.

3. World Health Organization. Worldwide prevalence of anemia 1993–2005. WHO Global Database on Anemia; 2008.

4. Central Statistical Agency [Ethiopia]. Ethiopia Demographic and Health Survey 2016. Addis Ababa, Ethiopia, and Rockville, Maryland; 2016.

5. World Health Organization. The global prevalence of anemia in 2011. Geneva: World Health Organization; 2015.

6. Magalhaes RJS, Clements AC. Spatial heterogeneity of haemoglobin concentration in preschool-age children in sub-Saharan Africa. Bull World Health Organ. 2011;89(6):459-68.

7. Central Statistical Agency [Ethiopia], ICF International. Ethiopia Demographic and Health Survey 2011. Addis Ababa, Ethiopia and Calverton, Maryland, USA. Central Statistical Agency and ICF International; 2012.

8. Federal Democratic Republic of Ethiopia. National Nutrition Programme June 2013-June 2015; 2015.

9. World Health Organization. Global nutrition targets 2025. anaemia policy brief (WHO/NMH/NHD/144) Geneva: World Health Organization; 2014.

10. Bernard J. Brabin ZP, Francine Verhoeff. An Analysis of Anemia and Child Mortality. The journal of Nutrition 2001;131(2):636-48.

11. Woldie H, Kebede Y, Tariku A. Factors Associated with Anemia among Children Aged 6–23 Months Attending Growth Monitoring at Tsitsika Health Center, Wag-Himra Zone, Northeast Ethiopia. Journal of Nutrition and Metabolism. 2015.

12. Jemal Y, Haidar J, Makau WK. The magnitude and determinants of anaemia among refugee preschool children from the Kebribeyah refugee camp, Somali region, Ethiopia. South African Journal of Clinical Nutrition. 2017;30(1):1-6.

13. Muchie KF. Determinants of severity level of anemia among children aged 6-59 months in Ethiopia: Further analysis of the 2011 Ethiopian Demographic and health survey. BMC Nutrition 2016;2(51).
14. Gebreegziabiher G, Etana B, Niggusie D. Determinants of Anemia among Children Aged 6–59 Months Living in Kilte Awulaelo Woreda, Northern Ethiopia. Hindawi Publishing Corporation.; 2014.
15. Gari T. Anaemia among children in a drought affected community in south-central Ethiopia. PLoS ONE. 2017;12(3).
16. Rosen G. A history of public health. Baltimore: Johns Hopkins University Press. 1993.
17. Heck RH, Thomas SL, Tabata LN. Multilevel and longitudinal modeling with IBM SPSS2010.
18. Kawo KN, Asfaw ZG, Sebro NY. Multilevel Analysis of Determinants of Anemia Prevalence among Children aged 6-59 Months in Ethiopia: Classical and Bayesian approaches. 2016.
19. Dey S, Raheem E. Multilevel multinomial logistic regression model for identifying factors associated with anemia in children 6–59 months in northeastern states of India. Cogent Mathematics. 2016.
20. Ngnie-Teta I, Receveur O, Defo BK, Receveur O. Risk factors for moderate to severe anemia among children in Benin and Mali: Insights from a multilevel analysis. Food and Nutrition Bulletin. 2007;28(1).
21. Ngwira A, Kazembe L. Analysis of severity of childhood anemia in Malawi: a Bayesian ordered categories model. Open Access Medical Statistics; 2016;6:9-20.
22. Stoltzfus JC. Logistic Regression: A Brief Primer. Academic Emergency Medicine.; 2011.
23. Ali S, Ali A, Khan SA, Hussain S. Sufficient Sample Size and Power in Multilevel Ordinal Logistic Regression Models. Computational and Mathematical Methods in Medicine; 2016.
24. Steenbergen MR. The Multilevel Logit Model for Binary Dependent Variables; 2012.
25. Hox JJ. Multilevel Analysis: Techniques and Applications second ed. Great Britain: Routledge; 2010.
26. Williams R. Multicollinearity. http://www3ndedu/william/; 2015.
27. Hosmer DW, Lemeshow S. Applied Logistic Regression. Second Edition ed; 2000.
28. Khan JR, Awan N, Misu F. Determinants of anemia among 6–59 months aged children in Bangladesh: evidence from nationally representative data. BMC Pediatrics. 2016;16(3).
29. Tengco L, Rayco-p S, Solon J, Sarol J, SAlon F. Determinants of anemia among preschool children in the philippines. J Am coll nutr 2008;27(2):229-43.

30. Ray S, Chandra J, Jayashree Bhattacharjee, Sharma S, Agarwala A. Determinants of nutritional anaemia in children less than five years age in New Delhi, India International Journal of Contemporary Pediatrics. 2016;3(2):403-8.

31. Singh RK, Patra S. Extent of Anaemia among Preschool Children in EAG States, India: A Chllenge to Policy Makers. Hindawi Publishing Corporation. 2014.

32. Allen L. Biological mechanisms that might underlie iron's effects on fetal growth and preterm birth. J Nutr. 2001;131(2):581–9.

33. Semba R, Pee Sd, Ricks M, Sari M, Bloem MW. Diarrhea and fever as risk factors for anemia among children under age five living in urban slum areas of Indonesia. International Journal of Infectious Diseases. 2007;12:62-70.

34. Gayawan E, Arogundadeb E, Adebayo S. Possible determinants and spatial patterns of anaemia among young children in Nigeria: a Bayesian semi-parametric modeling Int Health 2014;6:35–45.

35. Ai Zhao YZ, Ying Peng, Jiayin Li, Titi Yang, Zhaoyan Liu, Yanli Lv, Peiyu Wang. Prevalence of Anemia and Its Risk Factors Among Children 6–36 Months Old in Burma. 2012.

36. Quintero JP, Siqueira AM, Tobon A, Blair S, Mreno A, Herrera MA, et al. Malaria-related anemia: a Latin American perspective. Men Inst Oswaldo cruz. 2011;106:91-104.

37. Fosu MO, Frimpong FO, Arthur MO. Factors Associated with Haemoglobin Prevalence among Ghanaian Children Aged 6 – 59 months. Journal of Biology, Agriculture and Healthcare; 2014;4(2).

38. Kejo D, Petrucka PM, Martin H, Kimanya ME, Mosha TC. Prevalence and predictors of anemia among children under 5 years of age in Arusha District, Tanzania. Pediatric Health, Medicine and Therapeutics; 2018.

Supporting information

S1 File. Authorization letter.
(PDF)

S2 File. Child dataset final.
(DTA)

Annex 1: Random effects estimates of anemia for included children age 6-59 months selected from the 2016 (n=7790)

Estimates	Null mode	Model 1	Model 2	Model 3
Variance ($\delta^2 u_0$)	0.76*	0.36*	0.28*	0.25*
ICC (%)	18.77	10.03	8.04	7.3
PCV (%)	(ref)	52.6	63.2	67
Model comparison AIC	9967	8373.8	9692.9	8281

Key: AIC – Akaike Information Criteria, ICC - Intraclass Correlation Coefficient

PCV – Percentage Change in Variance,* p-value<0.001

1. WHO. The global prevalence of anemia in 2011.Geneva: World Health Organization. 2015.
2. Magalhaes RJS, Clements AC. Spatial heterogeneity of haemoglobin concentration in preschool-age children in sub-Saharan Africa. Bull World Health Organ. 2011;89(6):459-68
3. Central, Statistical, Agency, [Ethiopia], ICF, International. Ethiopia Demographic and Health Survey 2011. Addis Ababa, Ethiopia and Calverton, Maryland, USA. Central Statistical Agency and ICF International. 2012.
4. Central, Statistica, lAgency, [Ethiopia]. Ethiopia Demographic and Health Survey 2016. Addis Ababa, Ethiopia, and Rockville, Maryland, USA. CSA and ICF. 2016.
5. Federal, Democratic, Republic, of, Ethiopia. National Nutrition Programme June 2013-June 2015. 2015.
6. WHO. Global nutrition targets 2025. anaemia policy brief (WHO/NMH/NHD/144) Geneva: World Health Organization. 2014.
7. Bernard J. Brabin ZP, Francine Verhoeff. An Analysis of Anemia and Child Mortality. The journal of Nutrition 2001;131(2):636-48.

8. WHO. Worldwide prevalence of anemia 1993–2005: . WHO Global Database on Anemia Geneva, WHO

2008.
9. WHO. Haemoglobin concentrations for the diagnosis of anaemia and assessment of severity. Vitamin and Mineral Nutrition Information System Geneva, World Health Organization (WHO/NMH/NHD/MNM/111)

2011.
10. WHO. Iron Deficiency Anaemia Assessment, Prevention and Control

2001.
11. Woldie H, Kebede Y, Tariku A. Factors Associated with Anemia among Children Aged 6–23 Months Attending Growth Monitoring at Tsitsika Health Center, Wag-Himra Zone, Northeast Ethiopia. Journal of Nutrition and Metabolism. 2015.
12. Jemal Y, Haidar J, Makau WK. The magnitude and determinants of anaemia among refugee preschool children from the Kebribeyah refugee camp, Somali region, Ethiopia. South African Journal of Clinical Nutrition. 2017;30(1):1-6.
13. Muchie KF. Determinants of severity level of anemia among children aged 6-59 months in Ethiopia: Further analysis of the 2011 Ethiopian Demographic and health survey. BMC Nutrition 2016;2(51).
14. Gebreegziabiher G, Etana B, Niggusie D. Determinants of Anemia among Children Aged 6–59 Months Living in Kilte Awulaelo Woreda, Northern Ethiopia. Hindawi Publishing Corporation. 2014.
15. Gari T. Anaemia among children in a drought affected community in south-central Ethiopia. PLoS ONE. 2017.;12(3).
16. Rosen G. A history of public health. Baltimore: Johns Hopkins University Press. 1993.
17. Heck RH, Thomas SL, Tabata LN. Multilevel and longitudinal modeling with IBM SPSS2010.
18. Kawo KN, Asfaw ZG, Sebro NY. Multilevel Analysis of Determinants of Anemia Prevalence among Children aged 6-59 Months in Ethiopia: Classical and Bayesian approaches. 2016.
19. Dey S, Raheem E. Multilevel multinomial logistic regression model for identifying factors associated with anemia in children 6–59 months in northeastern states of India. Cogent Mathematics. 2016.
20. Ngnie-Teta I, Receveur O, Defo BK, Receveur O. Risk factors for moderate to severe anemia among children in Benin and Mali: Insights from a multilevel analysis. Food and Nutrition Bulletin. 2007;28(1).
21. Ngwira A, Kazembe L. Analysis of severity of childhood anemia in Malawi: a Bayesian ordered categories model

Open Access Medical Statistics. 2016.;6:9-20.
22. Stoltzfus JC. Logistic Regression: A Brief Primer. Academic Emergency Medicine. 2011.
23. Ali S, Ali A, Khan SA, Hussain S. Sufficient Sample Size and Power in Multilevel Ordinal Logistic Regression Models. Computational and Mathematical Methods in Medicine. 2016.
24. Steenbergen MR. The Multilevel Logit Model for Binary Dependent Variables. 2012.
25. Hox JJ. Multilevel Analysis: Techniques and Applications

second ed. Great Britain: Routledge; 2010.
26. Williams R. Multicollinearity. http://www3ndedu/william/. 2015.
27. Hosmer DW, Lemeshow S. Applied Logistic Regression. Second Edition ed2000.
28. Khan JR, Awan N, Misu F. Determinants of anemia among 6–59 months aged children in Bangladesh: evidence from nationally representative data. BMC Pediatrics. 2016;16(3).
29. Tengco L, Rayco-p S, Solon J, Sarol J, SAlon F. Determinants of anemia among preschool children in the philippines. J Am coll nutr 2008;27(2):229-43.
30. Ray S, Chandra J, Jayashree Bhattacharjee, Sharma S, Agarwala A. Determinants of nutritional anaemia in children less than five years age in New Delhi, India International Journal of Contemporary Pediatrics. 2016;3(2):403-8.
31. Singh RK, Patra S. Extent of Anaemia among Preschool Children in EAG States, India: A Challenge to Policy Makers. Hindawi Publishing Corporation. 2014.
32. Allen L. Biological mechanisms that might underlie iron's effects on fetal growth and preterm birth. J Nutr. 2001;131

(2):581–9.
33. Semba R, Pee Sd, Ricks M, Sari M, Bloem MW. Diarrhea and fever as risk factors for anemia among children under age five living in urban slum areas of Indonesia. International Journal of Infectious Diseases. 2007;12:62-70.
34. Gayawan E, Arogundadeb E, Adebayo S. Possible determinants and spatial patterns of anaemia among young children in Nigeria: a Bayesian semi-parametric modeling

Int Health 2014;6:35–45.
35. Ai Zhao YZ, Ying Peng, Jiayin Li, Titi Yang, Zhaoyan Liu, Yanli Lv, Peiyu Wang. Prevalence of Anemia and Its Risk Factors Among Children 6–36 Months Old in Burma. 2012.
36. Quintero JP, Siqueira AM, Tobon A, Blair S, Mreno A, Herrera MA, et al. Malaria-related anemia: a Latin American perspective. Men Inst Oswaldo cruz. 2011;106:91-104.
37. Fosu MO, Frimpong FO, Arthur MO. Factors Associated with Haemoglobin Prevalence among Ghanaian Children Aged 6 – 59 months. Journal of Biology, Agriculture and Healthcare. 2014;4(2).
38. Kejo D, Petrucka PM, Martin H, Kimanya ME, Mosha TC. Prevalence and predictors of anemia among children under 5 years of age in Arusha District, Tanzania. Pediatric Health, Medicine and Therapeutics. 2018.
